# Cryo-EM structures of autoinhibited and RNA-activated ZNFX1 helicase

**DOI:** 10.1038/s41467-026-78145-0

**Published:** 2026-09-25

**Authors:** Qing He, Feng Wang, Feyruz V. Rassool, Stephen B. Baylin, Huilin Li

**Affiliations:** 1 Department of Structural Biology, Van Andel Institute, Grand Rapids, MI USA; 2 University of Maryland Marlene and Stewart Greenebaum Comprehensive Cancer Center, Baltimore, MD USA; 3 Division of Translational Radiation Sciences, Department of Radiation Oncology, University of Maryland School of Medicine, Baltimore, MD USA; 4 Department of Oncology, Sidney Kimmel Comprehensive Cancer Center at Johns Hopkins, The Johns Hopkins University School of Medicine, Baltimore, MD USA

**Keywords:** Cryoelectron microscopy, Enzyme mechanisms

## Abstract

ZNFX1 is an RNA helicase that plays a critical role in host defense against viral infections. Recent studies further revealed that ZNFX1 harbors an unexpected RNA-ubiquitinating E3 ligase activity, raising fundamental questions about how RNA-binding activates this multifunctional enzyme. Here, we combine cryo-electron microscopy with biochemical analyses to define the structural basis of ZNFX1 regulation. In the absence of RNA, ZNFX1 adopts an autoinhibited conformation in which a blocking helix occludes the RNA-binding groove. It exists predominantly as autoinhibited monomers, and, at higher concentrations, assembles into short helical filaments composed of repeating tetramers. Binding of single-stranded RNA (ssRNA) remodels these higher-order assemblies into RNA-bound ZNFX1 monomers and dimers. The cryo-EM structure of the ssRNA-bound ZNFX1 dimer reveals that RNA binding displaces the blocking helix and induces large-scale conformational rearrangements that remodel the helicase core into an active configuration. Biochemical analyses validate the proposed RNA-binding interfaces and demonstrate ATP-dependent RNA unwinding by ZNFX1. Together, these findings support a regulatory mechanism in which autoinhibited ZNFX1 exists as monomers or concentration-dependent higher-order oligomers, whereas RNA binding promotes structural remodeling and helicase activation.

## Abstract (web summary)

ZNFX1 is an RNA helicase that plays a critical role in host defense against viral infections. Here, the authors reveal how RNA binding remodels autoinhibited ZNFX1 assemblies and activates the helicase for ATP-dependent RNA unwinding.

## Introduction

Innate immunity relies on a coordinated network of cellular and molecular pathways that rapidly detect invading pathogens and initiate early protective responses^1,2^. Central to this process is the ability to sense foreign nucleic acids while tightly regulating downstream inflammatory signaling to avoid excessive or inappropriate activation^2,3^. RNA helicases such as RIG-I, MDA5, and LGP2 play key roles in these pathways by coupling RNA recognition to ATP-dependent conformational changes and signal-amplifying oligomerization^4–8^.

ZNFX1 (NFX1-type zinc finger-containing 1) is an interferon-stimulated superfamily 1 (SF1) RNA helicase that has recently emerged as a key regulator of antiviral immunity and immune homeostasis^9–11^. ZNFX1 localizes to mitochondria, where it engages viral RNA and promotes antiviral signaling through MAVS^12,13^. Loss-of-function mutations in ZNFX1 cause recurrent viral and bacterial infections, immune dysregulation, and impaired cellular responses to nucleic-acid stress, establishing ZNFX1 as a critical determinant of host defense^14–17^. In addition, ZNFX1 participates in stress granule-associated pathways and syndromic susceptibility phenotypes^17–19^, highlighting its broad role in coordinating immune and stress responses across multiple cellular contexts^13^.

Beyond its role in antiviral signaling, ZNFX1 was recently shown to negatively regulate NLRP3 inflammasome activation, thereby restraining excessive inflammatory responses^15^. We further demonstrated that ZNFX1 acts as a master mediator of epigenetically induced pathogen mimicry, leading to mitochondrial dysfunction and activation of stimulator of interferon genes (STING), and functioning as a tumor suppressor^9^. Most strikingly, ZNFX1 was recently identified as an RNA-ubiquitinating E3 ligase that modifies and clusters single-stranded RNA into ubiquitin-coated ribonucleoprotein assemblies, revealing an unexpected RNA-centered regulatory function within the ubiquitin system^20–22^. These findings raise fundamental questions regarding how RNA-binding activates and coordinates its helicase and E3 ligase activities.

**Fig. 1:**
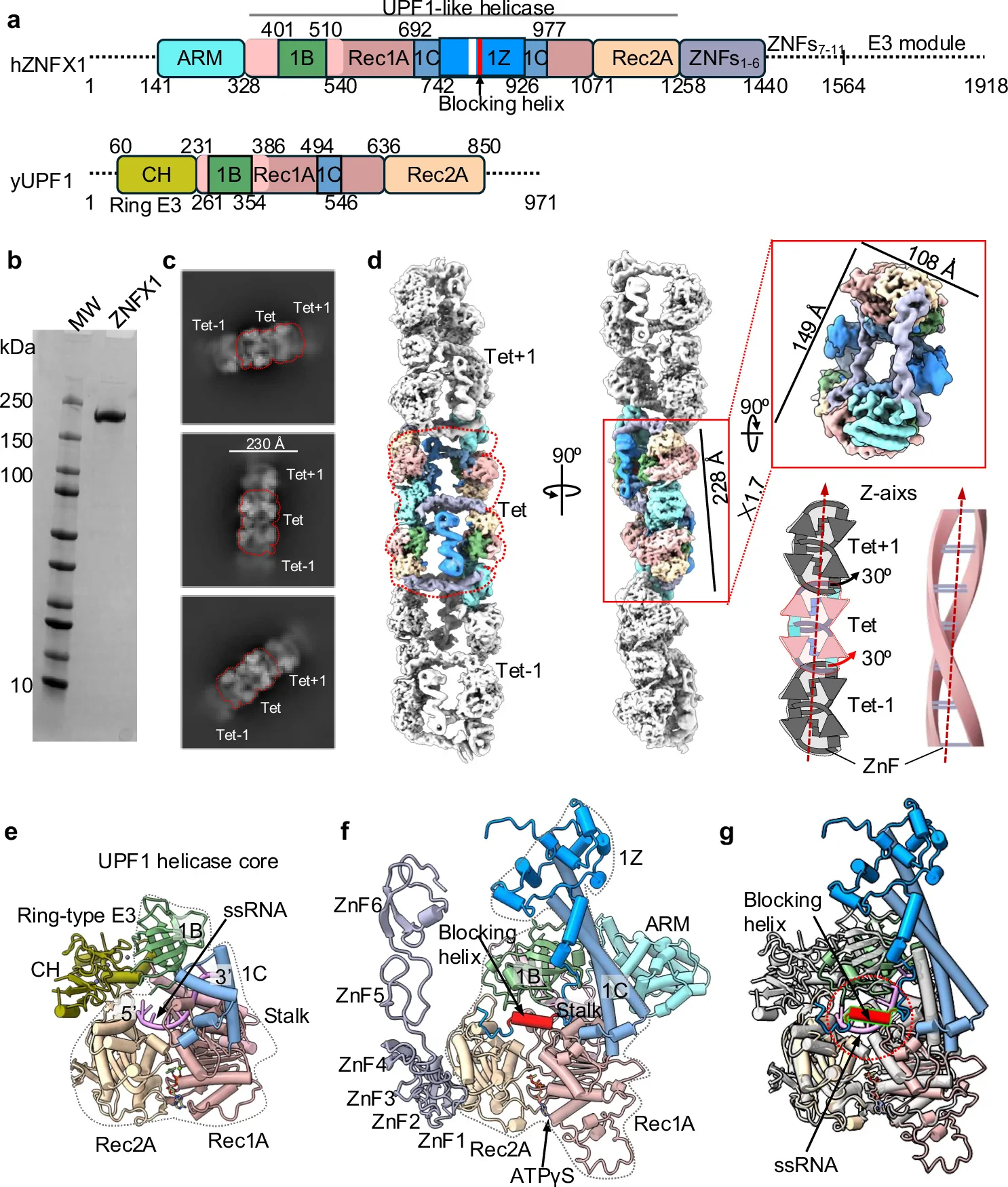
Cryo-EM structure of as-purified ZNFX1 forming a tetramer-based helical filament. **a** Domain organization of human ZNFX1 and the homologous yeast UPF1, highlighting the conserved helicase core and the ZNFX1-specific ARM, ZnF, and E3 modules. The unresolved N-terminal region, the C-terminal six ZnF motifs, and the E3 module are shown in gray. The blocking helix is indicated by a red bar. **b** SDS–PAGE analysis of purified ZNFX1. This experiment was independently repeated >3 times with similar results. **c** Representative 2D class averages of ZNFX1 particles showing a central tetramer flanked by neighboring tetramers, consistent with short filament formation. **d** Composite cryo-EM 3D map of a short helical filament comprising three ZNFX1 tetramers. The central tetramer is fully resolved, whereas the + 1 and – 1 tetramers were generated by docking the central tetramer into the corresponding densities. One protomer is colored by domain, and the remaining protomers are shown in different shades of gray. Upper right, enlarged view of the tetramer. Lower right, ~ 30° axial rotation between adjacent tetramers (left) and the resulting double-helical filament architecture (right). The left and right strands are each formed by the head-to-tail arrangement of side-by-side dimers that are laterally connected by the ZnF arms. **e** Structure of the UPF1 helicase core bound to ssRNA (PDB: 2XZL). **f** Structure of ZNFX1 protomer showing the conserved UPF1-like helicase core together with the additional ARM domain and ZnF arms. The helicase core and ARM were built into the cryo-EM density, whereas ZnF1–6 were modeled by fitting AlphaFold predictions into lower-resolution density. **g** Structural superposition of the helicase cores of ZNFX1 and UPF1. The blocking helix of ZNFX1 occupies the ssRNA-binding groove and sterically clashes with the ssRNA bound to UPF1, consistent with an autoinhibited conformation.

Human ZNFX1 is a large, multidomain enzyme of 1,918 amino acids with an estimated molecular mass of ~ 220 kDa. A previous cryo-EM structure of monomeric ZNFX1 revealed a UPF1-like helicase core (Rec1A and Rec2A), an N-terminal Armadillo (ARM) repeat domain, an expanded 1 C domain containing a unique 1Z insertion, a long arm comprising twelve zinc-finger (ZnF) motifs; the C-terminal E3 ligase module was not resolved^13,20^ (Fig. 1a). This domain organization suggests that ZNFX1 integrates RNA recognition with protein–protein interactions and ubiquitin signaling. However, because the structure was determined in the absence of nucleic acid, it likely represents an inactive state.

Additional evidence suggests that dimerization may be critical for ZNFX1 activation. A computational model of a ZNFX1 dimer based on the crystal structure of the truncated ARM domain, together with averaged EM images of negatively stained ssDNA-bound ZNFX1, revealed dimeric assemblies. AlphaFold predictions further indicated that the two key motifs of the E3 ligase module—the RZ and Z-ring—are spatially separated within a single ZNFX1 molecule but come together in the dimer, suggesting a trans-acting E3 ligase mechanism that requires ZNFX1 dimerization^20^. Despite these insights, the structural basis by which RNA binding remodels oligomeric states and activates the helicase remains unclear.

To address these questions, we perform a systematic cryo-EM analysis of full-length human ZNFX1 in distinct functional states, complemented by biochemical characterization. Here, we show that, in the absence of RNA, ZNFX1 adopts an autoinhibited conformation in which a blocking helix occludes the RNA-binding groove. Autoinhibited ZNFX1 exists as monomers and, in a concentration-dependent manner, assembles into short helical filaments composed of repeating tetramers. Binding of single-stranded RNA (ssRNA) remodels these higher-order assemblies into RNA-bound ZNFX1 monomers and dimers. Together with biochemical analyses of RNA binding, ATP hydrolysis, and RNA unwinding, our results support a regulatory mechanism in which RNA binding induces structural remodeling of ZNFX1 to promote helicase activation.

## Results

We recombinantly expressed and purified full-length human ZNFX1 in mammalian cells (Fig. 1b; “Methods”). During size-exclusion chromatography (SEC), purified ZNFX1 eluted at an apparent molecular weight substantially larger than that of a monomer, suggesting the presence of higher-order assemblies in solution (Supplementary Fig. 1a, b). Mass photometry showed that ZNFX1 was predominantly monomeric at ~ 20 nM, whereas increasing the protein concentration produced progressively broader mass distributions consistent with concentration-dependent self-association (Supplementary Fig. 2a). These observations agree with a recent study reporting concentration-dependent oligomerization of ZNFX1^22^. To define the structural basis of these higher-order assemblies and their remodeling by RNA, we next performed cryo-EM analysis.

### Tetramers constitute the repeating unit of ZNFX1 helical filaments

Cryo-EM analysis revealed that the higher-order assemblies are composed of repeating tetrameric units. Raw cryo-EM micrographs of purified ZNFX1 revealed a heterogeneous population of particles, including discrete small and large particles as well as short filamentous features (Supplementary Fig. 1b). Two-dimensional (2D) class averaging showed that the smaller particles corresponded to ZNFX1 monomers, whereas the larger particles represented tetramers (Supplementary Fig. 1c). Notably, many tetrameric class averages contained partial densities from neighboring tetramers, consistent with the short filaments (around 500-1000 Å) observed in the raw micrographs (Supplementary Fig. 1b). These observations agree with a recent study showing that ZNFX1 forms longer filaments and higher-order filament bundles at elevated protein concentrations^22^.

The monomeric particles exhibited severe preferred orientation, preventing reliable three-dimensional (3D) reconstruction. Because a cryo-EM structure of monomeric ZNFX1 has been reported previously^20^, we did not pursue further structural analysis of the monomeric particles. In contrast, the tetrameric particles sampled a substantially broader range of orientations (Supplementary Fig. 3a), enabling 3D reconstruction to an overall resolution of ~ 3.79 Å in C2 symmetry. Focused local refinement further improved the map to a final resolution of 3.71 Å in C1 symmetry. The resulting 3D map contained a fully-resolved central tetramer flanked by partial density corresponding to neighboring + 1 and – 1 tetramers. The fully resolved central tetramer (tetramer 0; dimensions of ~ 108 × 149 × 228 Å) was docked into the partial densities of the adjacent tetramers, yielding a composite cryo-EM map of a short helical filament comprising three tetramers (Fig. 1d and Supplementary Fig. 1d).

**Fig. 2:**
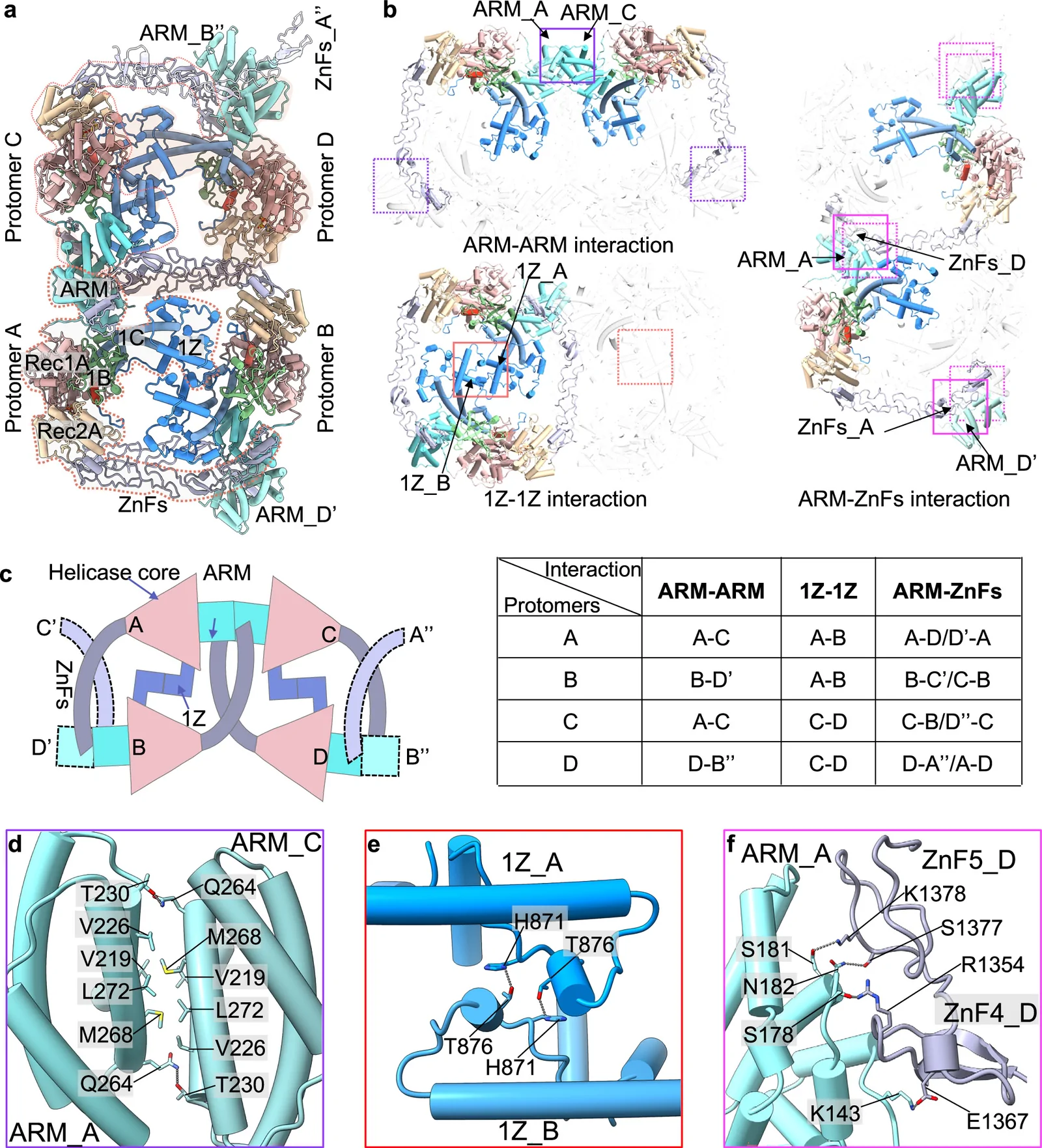
Interactions underlying ZNFX1 tetramerization and filament assembly. **a** Cartoon representation of the ZNFX1 tetramer, which serves as the repeating unit of the helical filament. Domains are colored as in Fig. 1a and labeled for protomer A. Interactions between adjacent tetramers are also indicated. **b** Three distinct inter-protomer interactions involving protomer A. The interacting protomers are shown in domain colors, while the remaining protomers are rendered in transparent gray. Protomers A and C form a side-by-side dimer through an ARM–ARM interaction (purple box); protomers A and B form a face-to-face dimer through a 1Z–1Z interaction (orange box); and protomers A and D form an S-shaped dimer through an ARM–ZnF interaction (magenta box). Equivalent interfaces are indicated by dashed boxes in the corresponding colors. **c** Schematic representation of the domain interactions within the ZNFX1 tetramer. Domains are colored individually for each protomer. The accompanying table summarizes the distribution of inter-domain contacts. **d**–**f** Close-up views of the ARM–ARM (**d**), 1Z–1Z (**e**), and ARM–ZnF (**f**) interfaces shown in panel (**b**). Interacting residues are shown as sticks. These interfaces are stabilized by complementary hydrophobic and polar interactions that collectively stabilize the tetrameric repeating unit and the helical filament.

The cryo-EM density was well resolved for the conserved ARM domain and helicase core, enabling atomic model building in these regions (Figs. 1e–f, 2a, Supplementary Fig. 3 and Supplementary Table 1). A ~ 300-amino-acid region between the helicase core and the E3 module is predicted to contain twelve zinc-finger (ZnF) motifs^20^. Of these, the first six ZnF motifs were partially stabilized within the tetramer but were resolved at lower local resolution, likely due to a combination of preferred orientation and conformational flexibility. An AlphaFold-predicted model of ZnF1–6 was docked into the corresponding density (Fig. 1f). In contrast, the N-terminal region (residues 1–140) and the C-terminal region containing ZnF7–12 and the E3 module (residues 1441–1918) were disordered and could not be modeled.

The ZNFX1 tetramer adopts a dimer-of-dimers organization, stabilized primarily via ARM–ARM interactions along the longitudinal direction and 1Z–1Z and ARM–ZnF interactions laterally. Adjacent tetramers are rotated by ~ 30° relative to one another along the filament axis, generating a twisted double-helical filament arrangement (Fig. 1d). In this organization, the left and right strands are composed of contiguously arranged side-by-side dimers formed via ARM–ARM interactions, whereas the two strands are laterally connected by ZnF-mediating and 1Z–1Z interactions. The interface between successive tetramers closely resembles that between the two dimers within a single tetramer, suggesting a conserved inter-tetramer interaction that could support filament extension (Fig. 1d). This interpretation is consistent with the formation of longer filaments and higher-order filament bundles at elevated protein concentrations reported recently^22^.

### A blocking helix maintains ZNFX1 in an autoinhibited conformation

Comparison of a ZNFX1 protomer extracted from the tetrameric assembly with the previously reported ssRNA-bound UPF1 structure^23^ revealed a conserved RecA-like helicase architecture, in which the canonical Rec1A and Rec2A domains form the ATPase and RNA-binding core (Fig. 1e, f). This structural similarity is consistent with prior classification of ZNFX1 as a UPF1-like SF1 RNA helicase^24^. However, several notable differences distinguish ZNFX1 from UPF1 and reveal structural features unique to ZNFX1.

Most prominently, the 1 C domain inserted within Rec1A is markedly expanded in ZNFX1 relative to UPF1, forming an elongated stalk capped by an additional helical insertion, termed the 1Z motif. This expansion suggests that the ZNFX1 1 C domain has evolved specialized regulatory and oligomerization roles beyond those of UPF1. In addition, ZNFX1 contains a large N-terminal ARM domain and multiple ZnF repeats that are absent in UPF1. These ZNFX1-specific domains extend outward from the helicase core and mediate extensive intermolecular interactions that stabilize higher-order assemblies. In contrast, the N-terminal CH domain of UPF1^25^ is positioned adjacent to the helicase core and is well ordered (Fig. 1e), whereas the C-terminal E3 ligase module of ZNFX1 is conformationally flexible and not resolved in the cryo-EM map (Fig. 1f). Nevertheless, recent AlphaFold predictions together with biochemical assays have defined this region as a split-site E3 ligase^20–22^.

Strikingly, an inspection of the ZNFX1 helicase core revealed that an α-helix originating from the 1Z domain occupies the single-strand nucleic acid-binding groove. The groove corresponds precisely to the ssRNA-binding site in UPF1 (Fig. 1g). Because the helix is enriched in negatively charged residues and sterically occludes the RNA-binding groove, we refer to it as the blocking helix. Its position within the substrate-binding groove indicates that ZNFX1 is locked in an autoinhibited conformation in the absence of RNA, preventing productive RNA engagement.

Our cryo-EM grids contain numerous monomeric ZNFX1 particles alongside ZNFX1 filaments (Supplementary Fig. 1c), indicating that monomeric ZNFX1 likewise adopts the autoinhibited conformation observed in tetrameric ZNFX1. This interpretation is consistent with a recent ZNFX1 study, which reported a monomeric, RNA-free structure in which the blocking helix remains bound within the RNA-binding groove^22^. Together, these observations indicate that autoinhibited ZNFX1 exists as both monomers and tetrameric/filamentous assemblies. The filamentous assemblies, therefore, likely represent concentration-dependent higher-order forms of the same autoinhibited monomeric state rather than a distinct autoinhibited conformation. The sequestration of the RNA-binding groove by the blocking helix, together with the inter-subunit interactions mediated by ZNFX1-specific domains, provides a structural basis for stabilizing both monomeric and filamentous ZNFX1 in an autoinhibited state prior to RNA engagement.

### Three interfaces stabilize ZNFX1 tetramers and filaments

To define the molecular interactions that drive ZNFX1 higher-order assembly, we analyzed the inter-subunit contacts within the tetrameric repeating unit and identified three distinct classes of interfaces involving the ARM domain, the 1Z insertion within the helicase 1 C domain, and the ZnF1–6 region (Fig. 2a). These interfaces collectively link individual ZNFX1 protomers into stable tetramers and further support assembly into short helical filaments ~ 500–1000 Å in length, corresponding to ~ 2–4 tetrameric repeats (Supplementary Fig. 1b).

Within the tetramer, protomers A and C form a side-by-side dimer through extensive ARM–ARM interactions, establishing the primary longitudinal scaffold of the assembly (Fig. 2b, left). Protomers A and B associate in a face-to-face orientation mediated by direct 1Z–1Z contacts near the helicase core (Fig. 2b, middle), whereas protomers A and D form an S-shaped dimer through interactions between the ARM domain of one protomer and the ZnF1–6 region of the other (Fig. 2b, right). Notably, protomer A also engages protomer D′ from an adjacent tetramer through the same ARM–ZnF interface, thereby linking adjacent tetramers along the filament axis.

A schematic representation illustrates how these three interfaces interconnect protomers both within and between tetramers to generate a continuous helical architecture (Fig. 2c, left). Quantitatively, each tetrameric repeat contains one internal and two shared ARM–ARM interfaces, two internal 1Z–1Z interfaces, and six ARM–ZnF interfaces (two internal and four shared with adjacent tetramers) (Fig. 2c, right). This extensive network of shared contacts provides a structural basis for ZNFX1 filament growth and stability.

The ARM–ARM interface forms the dominant stabilizing interaction within the tetramer (Fig. 2d). This interface is stabilized by complementary hydrophobic contacts involving Val219, Val226, Met268, and Leu272, as well as polar interactions contributed by Thr230 and Glu264. Together, these interactions promote the formation of stable side-by-side dimers that serve as the backbone for filament assembly.

The second interface is formed between the 1Z domains of adjacent protomers (Fig. 2e). This 1Z–1Z interface is positioned proximal to the helicase core and mediates face-to-face dimerization within the tetramer. Although the overall density for the 1Z domain was insufficient for unambiguous de novo modeling, the AlphaFold3-predicted model closely matched the density at the 1Z–1Z interface, in which His871 and Thr876 from opposing protomers are positioned to form symmetric hydrogen bonds (Fig. 2e). The presence of this interface suggests that expansion of the 1Z region within the ZNFX1 1 C domain may contribute to higher-order assembly of ZNFX1.

The third interface occurs between the ARM domain of one protomer and the ZnF1–6 region of the other, producing an S-shaped dimer configuration (Fig. 2f). The fitted model places ARM residues Lys143, Ser178, Ser181, and Asn182 near ZnF4–5 residues Arg1354, Glu1367, Ser1377, and Lys1378, suggesting possible charge–charge and polar interactions. However, owing to the limited local resolution, these specific side-chain contacts cannot be assigned unambiguously and should be regarded as model-based predictions.

Together, the 1Z–1Z interface and the ARM-ZnF interaction bridge the left and right strands formed by side-by-side dimers along the filament axis, reinforcing lateral connectivity and stabilizing the tetramer-based helical filament architecture.

### Cryo-EM structure of the RNA-bound ZNFX1 dimer

**Fig. 3:**
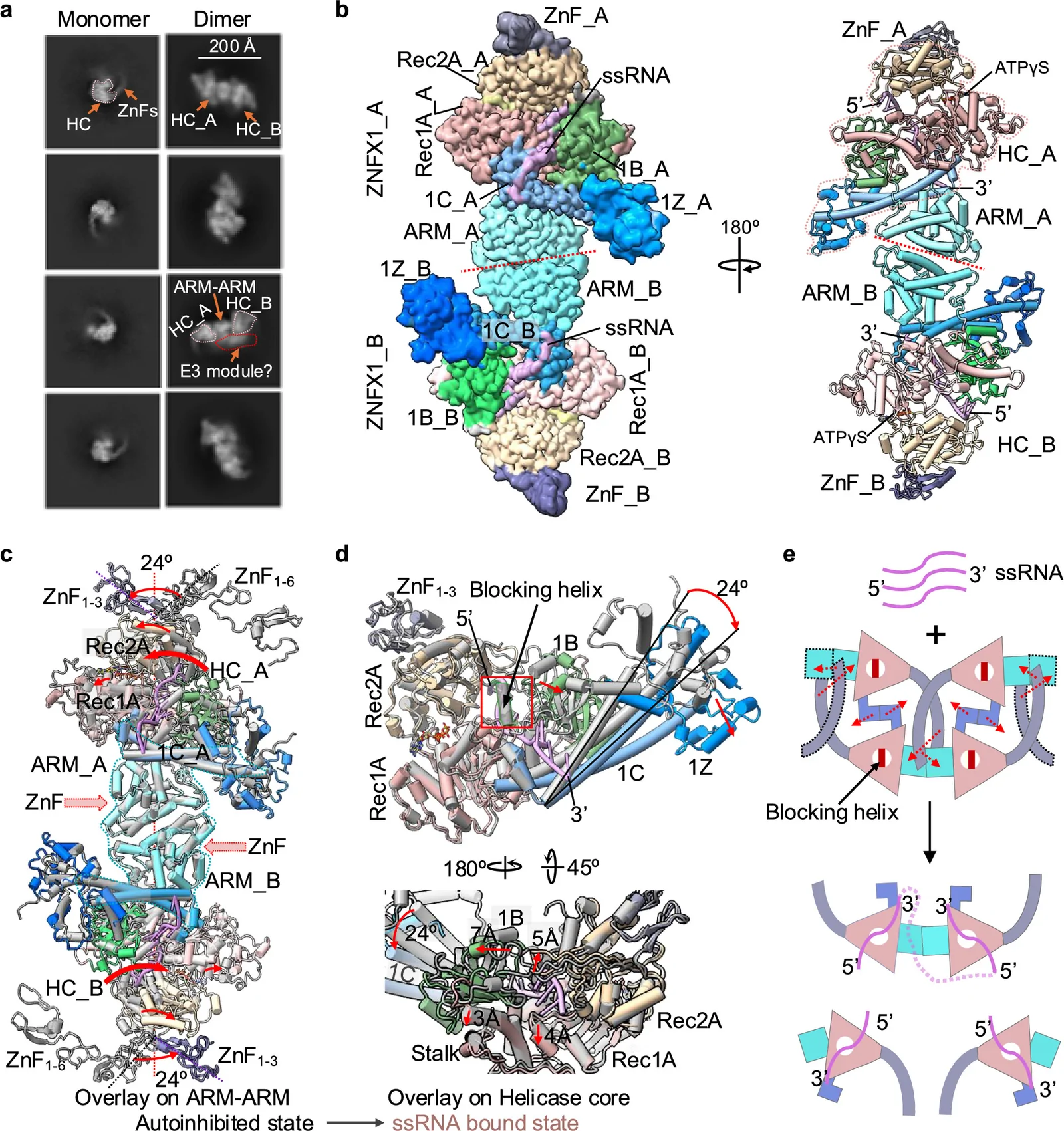
Cryo-EM structure of the ssRNA-bound ZNFX1 dimer. **a** Representative 2D class averages of ZNFX1 particles in the presence of ssRNA, showing monomeric (left) and dimeric assembly (right) particles. In monomeric particles, only the helicase core (HC) and the ZnF arm are well resolved. Dimeric particles exhibit additional weak density that may correspond to the flexible C-terminal E3 ligase module. **b** Cryo-EM 3D map (left) and atomic model (right) of the ssRNA-bound ZNFX1 dimer. Each protomer is colored by domain. The bound ssRNA is shown in purple, with the 5′ and 3′ ends indicated. **c** Structural superposition of the ssRNA-bound ZNFX1 dimer and a side-by-side dimer extracted from the autoinhibited filament, aligned using the ARM–ARM interface. Red arrows indicate an ~ 24° rotation of the ZnF linker region upon ssRNA binding. **d** Overlay of the ZNFX1 helicase core in the filament (gray) and ssRNA-bound (colored) states. Red arrows indicate an ssRNA-binding induced ~ 24° tilt of the 1C–1Z region. Bottom, conformational rearrangements within the helicase core that open the RNA-binding groove. **e** Model illustrating how the ssRNA-induced conformational changes in the ZnF linker region and 1Z domain remodel the higher-order ZNFX1 assemblies, yielding RNA-bound monomeric and dimeric species.

To determine how RNA binding remodels autoinhibited ZNFX1 into an active helicase, we next sought to determine the cryo-EM structure of ZNFX1 bound to single-stranded RNA (ssRNA), analogous to previously reported UPF1–RNA complexes^26^. Purified ZNFX1 was incubated with a 60-nt ssRNA substrate. Cryo-EM analysis revealed predominantly monomeric and dimeric particles, whereas tetrameric and filamentous assemblies were no longer observed (Fig. 3a). Consistent with the cryo-EM observations, mass photometry under the same conditions showed that ZNFX1 existed predominantly as monomers, with only a small population of dimers. When incubated with a shorter 15-nt ssRNA, ZNFX1 remained predominantly monomeric, whereas the proportion of dimers increased substantially with the 60-nt substrate (Supplementary Fig. 2b), indicating that longer ssRNA promotes formation of the RNA-bound dimer. Together, these cryo-EM and mass photometry analyses support a model in which longer ssRNA remodels the oligomeric state of ZNFX1 toward RNA-bound monomeric and dimeric species.

Although monomeric particles were abundant, they exhibited severe preferred orientation and did not yield a reliable three-dimensional reconstruction. The dimeric particles also retained some degree of orientation anisotropy but covered a sufficiently broad angular range to enable a 3D reconstruction to an overall resolution of 3.70 Å in C2 symmetry. Focused local refinement of a single monomer improved the map quality, increasing the overall resolution to 3.37 Å, enabling reliable atomic model building, particularly for the helicase core and dimerization interfaces (Fig. 3b, Supplementary Figs. 3, 4 and Supplementary Table 1). Moreover, the ARM–ARM dimer closely resembles the previously reported crystal structure of the ARM dimer (PDB 9EVE), with an RMSD of 0.875 Å over 286 Cα atoms (Supplementary Fig. 5), further supporting the reliability of the ARM mediated ZNFX1–ssRNA dimer model.

The resulting cryo-EM map reveals a symmetric ZNFX1 dimer bound to ssRNA, with each protomer engaging a continuous RNA segment (Fig. 3b). Because no density connects the two RNA segments, we cannot determine whether they originate from a single RNA molecule or from two independently bound RNA molecules. Two additional weak densities were observed in the 3D reconstruction: one located above the ARM domain and another extending from the 1Z domain toward the 1B domain and the RNA-binding cleft (Supplementary Fig. 6). These extra densities were also visible in representative 2D class averages of the dimer particles but were absent from the monomer particle classes (Fig. 3a). However, the local resolution of these densities was insufficient to support reliable model building. To further assess their identity, we attempted to dock the AlphaFold-predicted model of ZNFX1 into the low-threshold 3D map, but these weak densities do not correspond to the docked major helicase domain of ZNFX1 and cannot be confidently assigned to any previously characterized domains. Accordingly, we tentatively attribute these densities to the flexible C-terminal E3 ligase module. The approximate positioning of these features nonetheless suggests that the E3 ligase module may transiently associate with the helicase core and be influenced by RNA binding and dimer formation. Alternatively, as previous studies have shown that long nucleic acids can recruit multiple ZNFX1 molecules^20,21^, these weak densities may also arise from additional ZNFX1 protomers, potentially reflecting higher-order oligomeric assembly on ssRNA.

Overall, the cryo-EM structure of the ssRNA-bound ZNFX1 dimer, together with the accompanying mass photometry analyses, establishes a structural framework for understanding how RNA binding remodels autoinhibited ZNFX1 into catalytically competent monomeric and dimeric assemblies^20–22^.

### ssRNA binding remodels autoinhibited ZNFX1 assemblies

The ssRNA-bound ZNFX1 dimer closely resembles the side-by-side dimer observed within the autoinhibited tetramer and filament, with the same hydrophobic ARM–ARM interface mediating dimerization (Fig. 3b). In this configuration, each ZNFX1 subunit binds a continuous segment of ssRNA, with the RNA-binding groove oriented toward the central ARM–ARM interface. The RNA adopts an extended conformation, with its 5′ end deeply buried within the helicase core and the 3′ end extending toward the 1 C domain.

To understand how RNA binding remodels higher-order assemblies, we superimposed the ssRNA-bound dimer onto a side-by-side dimer extracted from the autoinhibited tetramer, using the largely invariant ARM–ARM interface for alignment. This comparison revealed that RNA binding induces an approximately 24° tilt of the ZnF linker region, accompanied by a smaller tilt of the helicase core relative to the ARM domain (Fig. 3c). Notably, the two protomers tilt in opposite directions.

In the context of the tetrameric and filamentous assemblies, these RNA-induced movements would destabilize both the ARM–ZnF interface and the ZnF-mediated contacts between adjacent tetramers along the filament axis. Consistent with this interpretation, the ZnF3–6 becomes poorly ordered in the ssRNA-bound state, indicating increased flexibility and loss of stabilizing inter-subunit contacts (Fig. 3c). In addition, the 1Z domain also becomes more flexible upon RNA binding, which may disrupt the 1Z–1Z interface. Thus, the RNA-bound conformation is incompatible with the ARM–ZnF and 1Z–1Z interactions that stabilize the tetrameric and filamentous assemblies.

Whereas the above comparison explains how RNA binding remodels higher-order assemblies, comparison of individual protomers reveals how RNA-binding activates the helicase. Further insight into the RNA-induced conformational changes was obtained by comparing individual ZNFX1 monomers from the autoinhibited and ssRNA-bound structures. Superposition of the helicase cores revealed coordinated rearrangements of the 1 C and 1Z regions upon ssRNA binding, with both domains undergoing an ~ 24° tilt relative to the Rec1A and Rec2A domains (Fig. 3d, top). Notably, ssRNA binding displaces the blocking helix that occupies the RNA-binding groove in the autoinhibited state, consistent with structural comparison to the UPF1–ssRNA complex (Figs. 1g, 3d).

Concomitant with these changes, the 1B domain shifts approximately 7.8 Å away from the largely stationary Rec2A domain, while the lower stalk region of Rec1A moves downward by ~ 3–4 Å (Fig. 3d, bottom). Together, these movements widen and deepen the RNA-binding groove, transforming it from a closed, autoinhibited configuration into an open conformation competent for RNA binding. These coordinated conformational changes provide a structural framework for understanding how RNA binding remodels autoinhibited ZNFX1 into a helicase-competent conformation (Fig. 3e).

In both the autoinhibited and ssRNA-bound structures, ATPγS is clearly resolved between the Rec1A and Rec2A domains (Supplementary Fig. 7). The nucleotide is coordinated by conserved residues, with Rec1A residues Lys625 and Thr626 and the Rec2A residues Arg1074 and Arg1223 forming hydrogen bonds with the ATPγS phosphate groups, while Tyr627 and His1073 stack with the adenine base (Supplementary Fig. 7a, b). Despite this conserved binding mode, comparison of the two states revealed subtle but reproducible differences in nucleotide coordination. In the ssRNA-bound structure, the catalytic dipeptide motif Glu1006-Glu1007 shifts by approximately 2–3 Å toward ATPγS, and the Rec1A residues Thr626 and Tyr627 also move closer to the nucleotide relative to the autoinhibited state (Supplementary Fig. 7c). These observations suggest that ATP is more loosely coordinated in the absence of RNA, potentially contributing to autoinhibition, whereas RNA binding stabilizes the ATPase active site in a configuration compatible with helicase activation.

These RNA-induced conformational changes establish a helicase-competent architecture, prompting us to investigate how ZNFX1 engages RNA substrates.

### An extended ssRNA–binding groove underlies ZNFX1 activation

**Fig. 4:**
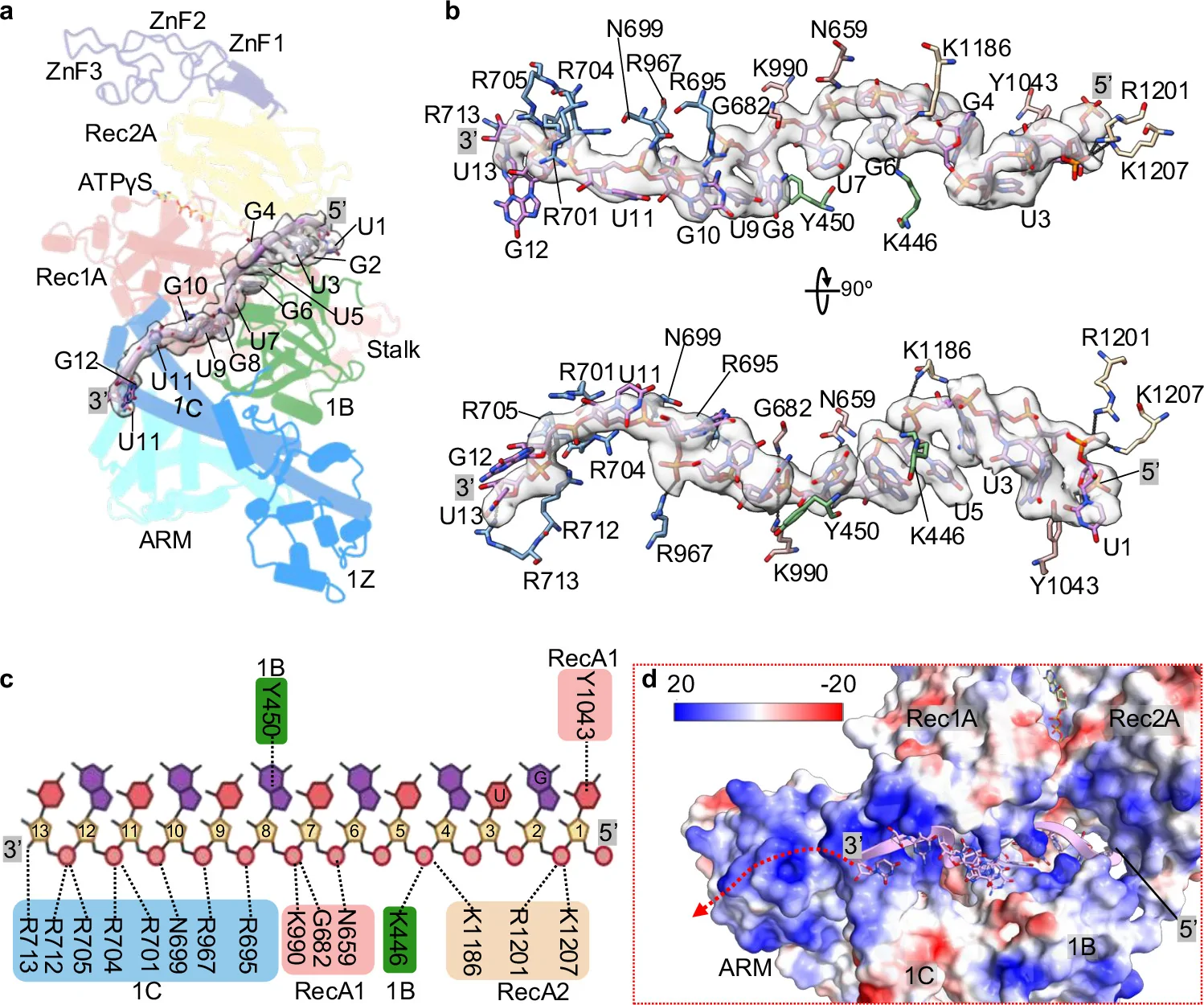
ssRNA recognition by ZNFX1. **a** Atomic model of ssRNA bound to the helicase core of one ZNFX1 protomer within the ARM-mediated dimer. The ssRNA is shown as sticks, with the corresponding cryo-EM density rendered transparently. Selected nucleotides are labeled to illustrate the RNA trajectory from the 3′ end toward the 5′ end through the helicase core. **b** Two views of the bound ssRNA showing the cryo-EM density, fitted atomic model, and interactions between the RNA and surrounding ZNFX1 residues along the extended RNA-binding groove. **c** Schematic summary of ssRNA interactions within the ZNFX1 helicase core. Positively charged and polar residues that contact the RNA backbone and bases are indicated, illustrating the distributed interaction network that stabilizes ssRNA binding. **d** Electrostatic surface representation of a ZNFX1 protomer showing a continuous positively charged groove that accommodates the ssRNA and extends from the 1 C domain toward the ARM domain. Electrostatic potentials were calculated in UCSF ChimeraX and are colored from red ( − 20 kT/e) to blue ( + 20 kT/e).

Thirteen ribonucleotides are well resolved and interact extensively with each ZNFX1 protomer (Fig. 4a–c). This represents a substantially longer RNA-binding footprint than observed in related SF1 helicases, where six and nine ribonucleotides are stabilized in UPF1 and IGHMBP2^23,27^ respectively (Supplementary Fig. 8). The RNA adopts an extended linear conformation within a positively charged groove (Fig. 4d), and RNA recognition is mediated almost exclusively through interactions with the phosphate backbone, supplemented by two π–π stacking interactions between tyrosine side chains and RNA bases (Fig. 4b, c). This mode of binding suggests that the helicase core recognizes ssRNA in a largely sequence-independent manner. This conclusion is consistent with previous studies showing that ZNFX1 binds a diverse range of RNA and DNA substrates both in vitro and in vivo^9,12,20^.

Within the helicase core, the Rec1A, Rec2A, and 1B domains collectively stabilize eight ribonucleotides located at the 5′ end and central region of the RNA (Fig. 4b, c). In this region, Lys1207 and Arg1201 contact the phosphate of G2, and Lys1186 contacts the phosphate of U5. In contrast, the Rec1A domain engages both the RNA backbone and bases: Asn659 hydrogen bonds with the phosphate of U7, Gly682 and Lys990 contact the phosphate of G8, and Tyr1043 forms a π–π stacking interaction with the U1 base. The 1B domain further contributes to RNA stabilization, with Lys446 reinforcing phosphate interactions near nucleotide U5 and Tyr450 stacking against the G8 nucleobase (Fig. 4b, c).

The remaining five ribonucleotides toward the 3′ end of the RNA are stabilized by the extended 1 C domain, a feature unique to ZNFX1 (Fig. 4a–c). Specifically, Arg695, Arg967, Asn699, Arg704, and Arg705 form hydrogen bonds with the phosphate groups of nucleotides U9, G10, U11, G12, and U13, respectively, while Arg701 contributes an additional hydrogen bond to the phosphate of G12. At the extreme 3′ end, Arg712 and Arg713 interact with the phosphate and ribose of nucleotide U13. In contrast, the 1 C domain of UPF1 does not engage ssRNA directly (Supplementary Fig. 8); instead, it stabilizes a structured histone stem–loop RNA^28^. In addition, the extended ssRNA-binding surface is highly conserved across species (Supplementary Fig. 9), supporting its functional importance. These extensive interactions explain how the helicase core engages ssRNA. We next investigated whether ZNFX1 contains an additional RNA-binding surface specialized for recognizing structured RNA.

### ZNFX1 contains both ssRNA- and dsRNA-binding regions

**Fig. 5:**
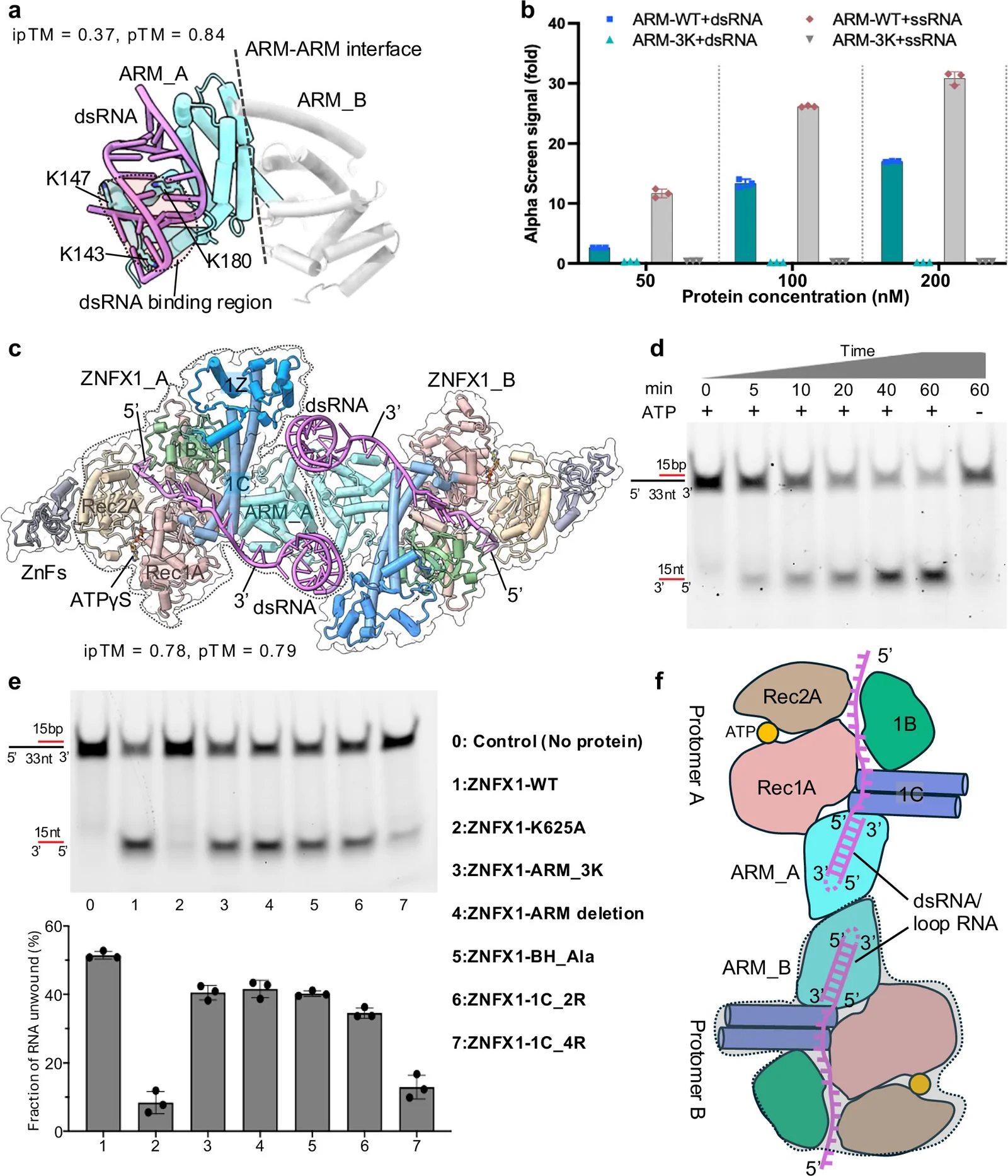
Mechanism of RNA unwinding by an ARM–ARM-mediated ZNFX1 dimer. **a** AlphaFold3-predicted ARM–dsRNA complex superimposed on one ARM protomer (gray) from the ssRNA-bound ZNFX1 dimer, showing that the positively charged dsRNA-binding surface is spatially distinct from the ARM–ARM dimerization interface. The predicted model confidence (pTM and ipTM scores) is indicated. **b** AlphaScreen analysis of RNA binding by the wild-type ARM domain and the ARM-3K mutant. Binding to both dsRNA and ssRNA substrates is shown. Data are presented as mean ± s.d. from three independent measurements. **c** Model of an ARM–ARM-mediated ZNFX1 dimer bound to RNA junction substrates. The model was generated by superimposing the AlphaFold3-predicted ZNFX1–RNA junction complex (outlined by the dashed line) onto each ARM domain of the cryo-EM dimer. The predicted model confidence (pTM and ipTM scores) is indicated. ZNFX1 is colored as in Fig. 3b, with domains labeled for protomer A. **d** Representative native gel showing ATP-dependent, time-dependent unwinding of the RNA junction substrate by wild-type ZNFX1. The duplex substrate and unwound RNA product are indicated. The experiments were independently repeated three times. **e** RNA-unwinding activities of wild-type ZNFX1 and the indicated mutants. A representative native PAGE gel is shown above, and the fraction of RNA unwound is quantified below. Data are presented as mean ± s.d. from three independent experiments. **f** Proposed model illustrating how the ARM–ARM-mediated ZNFX1 dimer simultaneously engages structured RNA through the ARM domains and adjacent ssRNA through the helicase core to promote duplex or stem-loop unwinding. Source data are provided as a Source Data file.

Whereas the helicase core and 1 C domain engage ssRNA, the ssRNA-bound ZNFX1 structure also reveals a broad, positively charged surface on the ARM domain that could mediate recognition of structured RNA (Fig. 4d). Although ARM domains are best known as protein–protein interaction modules, growing evidence indicates that tandem helical repeats can also engage nucleic acids through electrostatic interactions^29^. Consistent with this idea, the ZNFX1 ARM domain has been shown to bind viral double-stranded RNA (dsRNA) in cells^12^. To explore whether the ARM domain could directly engage structured RNA, we used AlphaFold3 to predict interactions between the ZNFX1 ARM domain and dsRNA. The resulting model predicts that dsRNA can bind to a cluster of positively charged residues on the ARM surface, including Lys143, Lys147, and Lys180 (Fig. 5a). Importantly, this predicted dsRNA-binding surface is spatially separated from the hydrophobic ARM–ARM dimerization interface (Fig. 5a), suggesting that dsRNA binding would be compatible with the ARM-mediated dimerization.

We experimentally tested the predicted ARM–RNA structure using an AlphaScreen assay (Amplified Luminescent Proximity Homogeneous Assay), which showed the ARM domain bound both dsRNA and ssRNA, with binding signals increasing in a protein concentration-dependent manner. Notably, the binding signal for ssRNA was approximately 2- to 4-fold stronger than that observed for dsRNA. In contrast, the ARM-3K mutant, in which Lys143, Lys147, and Lys180 were replaced with negatively charged glutamate residues, showed no detectable binding to either substrate, indicating that the positively charged surface of the ARM domain is essential for RNA binding (Fig. 5b).

We also used AlphaFold3 to predict the structure of a ZNFX1 monomer, comprising the ARM domain and helicase core, bound to an RNA junction substrate containing a dsRNA segment and a 5′ ssRNA overhang. The resulting model was predicted with high confidence (Fig. 5c). In this model, the dsRNA binds the same region of the ARM domain identified in the ARM–dsRNA structure (Fig. 5a, c), while the adjacent ssRNA segment extended toward the helicase core and 1 C domain (Fig. 5c). Together, the biochemical data and AlphaFold3 models support a proposed bipartite RNA-binding model in which each ZNFX1 subunit within the dimer could engage two distinct RNA elements: a single-stranded RNA segment bound by the helicase core and 1 C domain, and a structured RNA segment, such as a duplex or stem–loop, recognized by the ARM domain. This model suggests how ZNFX1 could simultaneously recognize structured RNA through the ARM domain and translocate along an adjacent ssRNA segment via the helicase core. Because each ARM domain is predicted to engage dsRNA independently, the ZNFX1 dimer could simultaneously engage two structured RNA elements (Fig. 5c).

### ZNFX1 unwinds dsRNA in an ATP-dependent manner

To determine whether ZNFX1 possesses RNA helicase activity, we performed unwinding assays using a fluorescently labeled RNA junction substrate. Wild-type ZNFX1 efficiently unwound the duplex in an ATP-dependent manner, and the extent of unwinding increased progressively over time (Fig. 5d). To our knowledge, this represents the first direct demonstration of RNA helicase activity for purified human ZNFX1.

To examine how the proposed bipartite RNA-binding architecture contributes to RNA unwinding, we generated seven ZNFX1 mutants targeting three functional modules: ATP hydrolysis (Walker A mutant K625A), RNA binding (ARM deletion, ARM_3K, a 1 C double-Arg mutant [R712A/R713A, 2 R], and a 1 C quadruple-Arg mutant [R695A/R698A/R701A/R704A, 4 R]), and the blocking helix (blocking-helix deletion, ΔBH, and blocking-helix alanine mutant, BH_Ala, in which all residues of the blocking helix were replaced with alanine). The ΔBH mutant was unstable during purification, yielding only a small amount of degraded protein (Supplementary Fig. 10). Although BH_Ala was expressed at high levels, most of the protein precipitated during size-exclusion chromatography, indicating that the blocking helix contributes to the structural integrity and stability of ZNFX1. Accordingly, only the BH_Ala mutant was used for subsequent biochemical analyses. Together, these mutants allowed us to evaluate the structural determinants of ATP hydrolysis, RNA binding, and blocking-helix-mediated autoinhibition.

We first compared the ATPase activities of wild-type ZNFX1 (ZNFX1-WT), the Walker A mutant (K625A), and the blocking-helix mutant (BH_Ala). In the absence of ssRNA, BH_Ala exhibited substantially higher basal ATPase activity than ZNFX1-WT. Addition of ssRNA stimulated the ATPase activity of ZNFX1-WT by approximately 2.2-fold, whereas the ATPase activity of BH_Ala increased by only 1.2-fold. As expected, the Walker A mutant K625A exhibited little detectable ATPase activity in either the absence or presence of ssRNA (Supplementary Fig. 11). These results indicate that the blocking helix suppresses basal ATPase activity in apo ZNFX1, whereas RNA binding relieves this inhibition in the wild-type protein.

We next compared the RNA-unwinding activities of ZNFX1-WT and the indicated mutants. ZNFX1-WT unwound approximately 51% of the RNA substrate, whereas the Walker A mutant K625A unwound only approximately 8% (Fig. 5e), confirming that ATP hydrolysis is essential for RNA helicase activity. ARM deletion and the ARM_3K mutation modestly reduced RNA unwinding to approximately 42% and 40%, respectively (Fig. 5e), indicating that the ARM domain and its positively charged RNA-binding surface contribute to efficient unwinding but are not essential for activity with the short junction substrate used here. Likewise, mutations of the positively charged RNA-binding surface within the 1 C domain also impaired RNA unwinding: the 1 C_2R and 1C_4R mutants unwound approximately 35% and 13% of the substrate, respectively, demonstrating that the extended ssRNA-binding surface of the 1C domain contributes to helicase activity. Finally, the BH_Ala mutant unwound approximately 40% of the RNA substrate (Fig. 5e), despite exhibiting elevated basal ATPase activity relative to ZNFX1-WT (Supplementary Fig. 11), indicating that increasing ATP hydrolysis alone is insufficient for efficient RNA unwinding. Together, these biochemical analyses are consistent with the bipartite RNA-binding model derived from the structural analyses and suggest that coordinated ATP hydrolysis and RNA engagement involving both the ARM domain and the helicase core contribute to efficient RNA unwinding.

### A model for structured RNA unwinding by ZNFX1

Together, the structural and biochemical data presented above support the following model in which the ARM domain may engage a structured RNA element, whereas the helicase core binds and translocates along an adjacent single-stranded RNA segment in an ATP-dependent manner, thereby promoting duplex or stem-loop unwinding (Fig. 5f). In this model, the ARM domain could stabilize the structured RNA substrate while the helicase core translocates along the neighboring ssRNA. A similar division of labor has been proposed for the Senataxin/Sen1 helicase, which contains a UPF1-like helicase core together with an N-terminal helical repeat domain that stabilizes RNA-DNA hybrids during unwinding^30^. Collectively, these structural and biochemical findings provide a mechanistic framework for understanding how ZNFX1 may couple structured RNA recognition with ATP-dependent RNA translocation and unwinding.

## Discussion

Here, we show that apo ZNFX1 adopts a blocking helix-mediated autoinhibited conformation that exists both as monomers and, in a concentration-dependent manner, as tetramers and short helical filaments. RNA binding remodels these higher-order assemblies into RNA-bound monomeric and dimeric states while simultaneously inducing conformational changes within the helicase core that promote helicase activation.

The autoinhibited ZNFX1 filament is organized through multiple inter-subunit interfaces (Fig. 2). The ARM–ARM interface is well resolved and independently supported by the reported ARM dimer crystal structure^20^ (Supplementary Fig. 5), whereas the density-supported 1Z–1Z interface was modeled with AlphaFold3 predicted structure, and the ARM–ZnF interface remains putative. Notably, most residues mediating the ARM–ARM and several residues positioned at the modeled 1Z–1Z interface are conserved across ZNFX1 homologs, whereas residues assigned to the putative ARM–ZnF interface show more limited conservation, suggesting that the proposed charged-charged interactions at this modeled interface should be interpreted with caution (Supplementary Fig. 9). These features are absent from other characterized UPF1-like helicases and collectively provide the structural basis for higher-order assembly unique among characterized UPF1-family helicases. In contrast, UPF1 adopts an autoinhibited conformation through intramolecular interactions between its N-terminal CH domain and the regulatory loop of 1B domain^31^.

The filamentous assemblies likely represent concentration-dependent higher-order forms of the same blocking-helix-mediated autoinhibited monomeric state rather than a distinct autoinhibited conformation. Such assemblies may serve as storage forms of ZNFX1 that can be rapidly remodeled into RNA-bound monomeric and dimeric species upon RNA engagement. These observations indicate that autoinhibition is mediated primarily by the blocking helix, whereas higher-order assembly represents a concentration-dependent organizational state.

During preparation of this manuscript, a preprint describing the cryo-EM structures of ZNFX1 filaments and an RNA-activated ZNFX1 monomer appeared in bioRxiv^22^. That study is consistent with our cryo-EM and mass photometry analyses demonstrating concentration-dependent oligomerization. Our work further defines the tetramer as the repeating unit of the filament and explains how RNA remodels these assemblies.

Both RNA-bound ZNFX1 monomer and dimer are competent to bind and unwind structured RNA substrates, raising the question of why ZNFX1 undergoes dimerization upon RNA engagement. A compelling explanation derives from the recently described split-site E3 ligase activity of ZNFX1, in which key catalytic elements that are spatially separated within a single protomer are proposed to assemble in trans upon dimer formation^20^. The RNA-activated dimer structure, therefore, provides a structural framework for this split-site mechanism and rationalizes the functional significance of RNA-induced dimerization.

Although the C-terminal E3 ligase module was not sufficiently ordered for atomic modeling, weak densities observed adjacent to the ARM and 1Z domains in the dimer structure suggest that this module may transiently associate with the helicase core (Fig. 3a and Supplementary Fig. 6). Stabilization of the E3 ligase module, potentially through binding of E2 enzyme, ubiquitin, or RNA substrates, will be an important goal for future structural studies aimed at understanding how ZNFX1 integrates helicase and ubiquitin ligase activities.

Finally, ZNFX1 possesses an unusually extended ssRNA-binding groove that spans the helicase core and the expanded 1 C domain, stabilizing thirteen ribonucleotides (Fig. 4). Together with a broad, positively charged RNA-binding surface on the ARM domain, this architecture supports a bipartite RNA-binding mode in which the ZNFX1 dimer can simultaneously engage single-stranded and structured RNA elements. Consistent with this model, biochemical analyses demonstrate that efficient RNA unwinding requires both the ARM domain and the 1 C RNA-binding surface, and disruption of the blocking helix increases basal ATPase activity while reducing RNA helicase activity (Fig. 5e). These findings support a model in which the ARM domain engages structured RNA while the helicase core binds and translocates along an adjacent ssRNA in an ATP-dependent manner, thereby promoting duplex and stem-loop unwinding. The proposed mechanism is reminiscent of the Senataxin/Sen1 helicase, which similarly couples ssRNA translocation with duplex capture via an N-terminal helical repeat domain^30^.

Because ZNFX1 is recognized as a master regulator of epigenetically driven pathogen-like immune responses and functions as a tumor suppressor in cancer^9^, its RNA-induced conformational switching may present therapeutic opportunities. Small molecules, nucleic acid mimetics, or engineered RNA ligands that stabilize the active dimeric conformation could, in principle, enhance ZNFX1 activity in contexts where its expression or function is limiting. Notably, analysis of The Cancer Genome Atlas (TCGA) datasets shows that ZNFX1 expression is significantly reduced in lung adenocarcinoma (LUAD) and lung squamous cell carcinoma (LUSC), the two major subtypes of non-small cell lung cancer (NSCLC), compared with normal lung tissue^9^. These malignancies may therefore be particularly amenable to strategies that restore or potentiate ZNFX1 activity. Our structural framework provides a foundation for the rational design of approaches to modulate ZNFX1 activation.

In summary, our work reveals a regulatory mechanism in which autoinhibited ZNFX1 exists as monomers and concentration-dependent filamentous assemblies. RNA binding remodels these assemblies into helicase-competent monomeric and dimeric states. Together, the structural and biochemical findings provide a mechanistic framework for understanding how ZNFX1 recognizes and unwinds structured RNA substrates while laying the foundation for future studies of its RNA-ubiquitinating E3 ligase activity.

## Methods

### DNA constructs and reagents

The human ZNFX1 gene was amplified from a plasmid purchased from the DNASU Plasmid Repository (HsCD00867565) and cloned into the pEG BacMam vector with an N-terminal 3xFLAG tag for mammalian expression. The same 3×FLAG-tagged ZNFX1 coding sequence was subsequently subcloned into the pFastBac Dual vector (Thermo Fisher Scientific) for insect-cell expression. ZNFX1 mutants were generated from the pFastBac-3×FLAG-ZNFX1 construct by PCR-based site-directed mutagenesis or overlap-extension PCR. The coding sequences for the ARM domain (residues 141–321) and the ARM-3K mutant were codon-optimized for expression in *Escherichia coli* and cloned into pET-28a with an N-terminal 6 × His tag. All constructs were verified by DNA sequencing.

ATPγS (adenosine 5′-O-(3-thio)triphosphate tetralithium salt; Sigma-Aldrich, Cat. No. 119120) was obtained from Sigma-Aldrich. Sf9 cells (Gibco, Cat. No. 11496015) and the FreeStyle^TM^ 293-F cells (Thermo Fisher Scientific, Cat. No. R79007) were obtained from Thermo Fisher Scientific.

### RNA oligonucleotides and substrate preparation

RNA oligonucleotides were synthesized by Integrated DNA Technologies (IDT). These included a 60-nt ssRNA (5’-UGUGUGUGUGUGUGUGUGUGUGUGUGUGUGUGUGUGUGUGUGUGUGUGUGUGUGUGUGUG-3’), a 33-nt biotin-labeled forward RNA (5’-biotin-GACUCAGUCACUGACAGCGAGUGCUACGACUAC-3’), a complementary 33-nt reverse RNA (5’-GUAGUCGUAGCACUCGCUGUCAGUGACUGAGUC-3’), a 33-nt junction forward RNA (5’- AUGCACUUGACGUACUAAAGCUAGUCAUGAGCU-3’), a 15-nt FAM-labeled junction reverse RNA (5’-FAM-AGCUCAUGACUAGCA-3’), and a 15-nt unlabeled trap RNA (5’-AGCUCAUGACUAGCA-3’).

For AlphaScreen assays, the dsRNA substrate was prepared by annealing equimolar amounts of the 33-nt biotin-labeled forward and the complementary reverse RNA to a final concentration of 2 μM in annealing buffer (10 mM HEPES, pH 7.5, 50 mM NaCl, and 1 mM EDTA). For helicase assays, the RNA junction substrate was prepared by annealing the 33-nt junction forward RNA and the 15-nt FAM-labeled junction reverse RNA at a molar ratio of 1.5:1 under the same conditions. RNA mixtures were heated to 95 °C for 3 min and gradually cooled to room temperature to allow duplex formation. Annealed substrates were stored at −20 °C until use.

### Protein expression and purification

Cryo-EM samples were prepared from mammalian-expressed ZNFX1, whereas biochemical assays used insect-cell-expressed protein to facilitate production of multiple mutant proteins.

For mammalian cell expression, the pEG-BacMam-ZNFX1 plasmid encoding human ZNFX1 with an N-terminal 3×FLAG tag was transformed into DH10Bac cells to generate bacmid DNA. Purified bacmid DNA was transfected into Sf9 cells to produce P1 and P2 baculoviruses^32^. P2 virus was harvested and used to infect FreeStyle™ 293-F cells (Thermo Fisher Scientific), which were cultured in FreeStyle™ 293 Expression Medium supplemented with 1% fetal bovine serum under 8% CO_2_. Suspension cultures at 2 –3 × 10^6^ cells/mL were infected with P2 virus (5% v/v) and incubated at 37 °C for 8–16 h. Sodium butyrate was then added to a final concentration of 7 mM, and cultures were shifted to 30 °C for protein expression.

ZNFX1, used for cryo-EM studies, was expressed in HEK293 cells as described above. Cells were harvested and resuspended in lysis buffer containing 25 mM HEPES (pH 7.5), 200 mM NaCl, 10% glycerol, and EDTA-free protease inhibitors. Lysates were prepared by sonication and clarified by ultracentrifugation (Ti-45 rotor, 40,000 rpm, 1 h, 4 °C). The supernatant was incubated with anti-FLAG affinity resin for 2–3 h at 4 °C, washed with 100 column volumes of lysis buffer, and eluted with lysis buffer supplemented with 0.2 mg/mL 3 × FLAG peptide. Eluted protein was concentrated using a 100 kDa molecular-weight-cutoff concentrator and further purified by size-exclusion chromatography on a Superose 6 Increase column (GE Healthcare) equilibrated in 25 mM HEPES (pH 7.5), 100 mM NaCl, and 0.5 mM TCEP. Peak fractions were pooled, concentrated, flash-frozen, and stored at − 80 °C.

For biochemical assays, wild-type ZNFX1 and the indicated mutants were expressed in Sf9 cells following the protocol described previously^20^. Briefly, Sf9 cells were infected with P2 baculoviruses (5% v/v), cultured for 48 h at 27 °C, and then incubated at 20 °C for an additional 24 h with constant shaking at 115 rpm. Cells were harvested 72 h after infection, lysed, and clarified. ZNFX1 proteins were purified by anti-FLAG affinity chromatography followed by size-exclusion chromatography using the same procedure described above. Purified proteins were concentrated, flash-frozen, stored at − 80 °C, and quantified using the Bradford assay.

For ARM domain purification, pET-28a plasmids encoding the ARM domain or ARM-3K mutant were transformed into E*scherichia coli* BL21 (DE3) cells. Cultures were grown in 2 L LB medium at 37 °C to an OD_600_ of approximately 0.6, induced with 0.2 mM IPTG, and incubated for an additional 12 h at 16 °C. Cells were harvested, resuspended in lysis buffer (25 mM HEPES, pH 7.6, 150 mM NaCl, and 10% glycerol) and lysed using a homogenizer (SPX Corporation). Lysates were clarified by centrifugation (20,000 × *g*, 40 min), and the supernatant was applied to a Ni–NTA affinity column. Bound protein was eluted with imidazole and further purified by size-exclusion chromatography on a Superdex 200 column (GE Healthcare) equilibrated in the same buffer.

### Mass photometry

Mass photometry measurements were performed using a OneMP instrument (Refeyn Ltd.) in buffer containing 25 mM HEPES (pH 7.5) and 150 mM KCl. ZNFX1 was analyzed at final concentrations ranging of 20 or 50 nM. For RNA-binding measurements, 20 nM ZNFX1 was incubated with 100 nM of either 15-nt or 60-nt ssRNA in the presence of 0.5 mM ATPγS. Movies were acquired for 1 min using the DiscoveryMP software. Particle mass distributions were analyzed with AnalysisMP (Refeyn) by fitting event histograms with Gaussian functions to determine the mean molecular masses.

### Electron microscopy sample preparation and data collection

For the apo ZNFX1 sample, purified ZNFX1 (1 µM) was incubated with 1 mM ATPγS at 4 °C for 30 min. For the ssRNA-bound complex, 1 µM ZNFX1 was mixed with 10 µM 60-nt ssRNA in the presence of 1 mM ATPγS, incubated at room temperature for 10 min, and then on ice for 2–3 h before grid preparation. The same preparation of purified ZNFX1 was used for both the apo and ssRNA-bound cryo-EM datasets.

In both cases, the holey carbon grids were glow-discharged in the Ar/O_2_ mixture for 30 s using a Gatan 950 Solarus plasma cleaning system, and a 3 µL sample was applied to freshly glow-discharged grids (Quantifoil R 1.2/1.3 Gold, 300 mesh). The grids were blotted for 3 seconds at a blot force 3 using qualitative cellulose filter paper (TED PELLA, INC), and plunge-frozen in liquid ethane cooled by liquid nitrogen. Grids were prepared using a Vitrobot Mark IV (Thermo Fisher Scientific) at 100% humidity and 5 °C. The cryo-EM grids were stored in liquid nitrogen until data collection.

Cryo-EM data were collected using a Titan Krios electron microscope (Thermo Fisher Scientific) operating at 300 kV and equipped with a Gatan K3 Summit direct electron detector. All images were recorded using SerialEM^33^, using a defocus range of – 1.2 to – 1.8 μm at a nominal magnification of 105,000 ×. The K3 detector was operated in super-resolution counting mode, resulting in a physical pixel size of 0.414 Å (binned to 0.828 Å for processing). Each movie consists of 50 frames with a total dose of 50 e^−^ /Å^2^.

### Image processing

#### Apo ZNFX1

A total of 40,862 movie stacks were collected for the autoinhibited apo ZNFX1 sample. Movies were motion-corrected using MotionCorr 2.0 with 2 × binning^34^ and imported into cryoSPARC (v4.7.1) for patch-based contrast transfer function (CTF) estimation^35^. A total of 34,460 micrographs with CTF fits better than 4.0 Å were retained for further processing (Supplementary Fig. 1d).

Initial blob-based particle picking identified both monomeric and tetrameric particles. Monomeric particles exhibited severe preferred orientation and were therefore excluded from further structural analysis. Blob-based particle picking (200–300 Å diameter) followed by template-based particle picking^35^ yielded 2,232,061 particles. After multiple rounds of 2D classification, 457,813 tetrameric particles (box size, 400 pixels) were selected for 3D reconstruction. Initial models were generated in C1 symmetry, followed by heterogeneous refinement to separate intact tetramers from poorly resolved classes. The best particle set was subjected to homogeneous and non-uniform refinements, yielding a 3D map at 3.89 Å resolution with C2 symmetry. Bayesian polishing was subsequently performed in Relion 5.0^36^, and the polished particles were transferred back to cryoSPARC for additional homogeneous and non-uniform refinement, improving the resolution to 3.79 Å.

To improve the local map quality, particle symmetry expansion and subtraction were performed, followed by local refinement of one half of the tetramer in C1 symmetry, resulting in a 3.71 Å reconstruction. The refined dimer map was then docked twice into the consensus C2 symmetry tetramer map, to generate the final composite reconstruction at 3.71 Å resolution.

#### ssRNA-bound ZNFX1

A total of 32,622 movie stacks were collected for the ssRNA-bound ZNFX1 complex and processed following the same workflow described above. After motion correction and patch-based CTF estimation in cryoSPARC (v4.7.1), 27,275 micrographs with CTF fits better than 4.0 Å were retained. Template-based particle picking identified 4,186,618 particles, which were subjected to multiple rounds of 2D classification. Well-defined particles were separated into monomeric and dimeric populations.

The monomeric subset (307,166 particles; box size, 360 pixels) exhibited severe preferred orientation and did not yield a reliable 3D reconstruction. The dimeric subset (165,198 particles; box size, 440 pixels) was used to generate initial 3D models in C2 symmetry. Homogeneous and non-uniform refinement produced a map at 3.75 Å resolution, followed by Bayesian polishing in RELION 5.0^36^ and additional homogeneous and non-uniform refinement in cryoSPARC, improving the overall resolution to 3.70 Å.

Following particle symmetry expansion and subtraction, local refinement of one ZNFX1-ssRNA protomer was performed in C1symmetry using a soft-edged mask, yielding a reconstruction at 3.37 Å resolution. The refined monomer map was then docked twice into the consensus dimer map to generate the final composite map at 3.37 Å resolution.

### Atomic model building and refinement

Initial atomic models were generated by docking the AlphaFold3-predicted structure of full-length human ZNFX1 into the final cryo-EM maps^37^. Individual domains, including the ARM, Rec1A, 1B, 1 C, Rec2A, and ZnF regions, were separated and independently fitted into the density using UCSF ChimeraX^38^. ATPγS molecules were manually placed into the nucleotide-binding pockets and refined in real space using Coot (v0.9.8.95)^39^. The EMReady (2.0)^40^-sharpened cryo-EM maps were used to facilitate model building.

For the autoinhibited apo structure, the cryo-EM density of 1Z was insufficient for unambiguous modeling. Therefore, AlphaFold3^37^ was used to predict the 1Z and 1Z-1Z interface, and the resulting model aligned well with the available cryo-EM density, with particularly close agreement at the 1Z–1Z interface. For the ssRNA-bound structure, the RNA density was of sufficient quality to allow manual model building, with clear discrimination of uridine and guanosine bases.

The models were manually adjusted in Coot and subjected to real-space refinement in Phenix^41^. Final models were validated using Molprobity^42^. Statistics of 3D reconstruction and model refinement are summarized in Supplementary Table 1. Structural figures were prepared using UCSF ChimeraX^38^.

### AlphaScreen assay

AlphaScreen assays were performed using the AlphaScreen His Detection Kit (PerkinElmer) according to the manufacturer’s instructions. Purified 6 × His-tagged ARM or ARM-3K (50–200 nM) were individually incubated with 50 nM biotinylated 33-bp dsRNA or 33-nt ssRNA in assay buffer containing 50 mM MOPS (pH 7.4), 100 mM NaCl, 0.005% CHAPS, and 0.1 mg/ml bovine serum albumin. Streptavidin-coated donor beads (5 μg/ml) and nickel-chelated acceptor beads (5 μg/ml) were added, and the mixture was incubated on ice for 1.5 h. AlphaScreen signals were measured with excitation at 680 nm and emission collected between 520 and 620 nm.

### RNA unwinding assay

RNA unwinding assays were performed using a FAM-labeled RNA junction substrate (10 nM) incubated with wild-type ZNFX1 or mutant ZNFX1 proteins (50 nM) in 10 µl of reaction buffer containing 20 mM HEPES (pH 7.0), 75 mM KCl, 5 mM MgCl₂, 1 mM DTT, 0.01% (v/v) Tween-20, and 1 µl of RNase inhibitor (20 U/µl). Reactions were preincubated for 15 min at room temperature before unwinding was initiated by the addition of an equal volume of reaction buffer containing ATP and trap RNA, yielding final concentrations of 4 mM ATP and 100 nM trap RNA. Control reactions lacking ATP were performed under identical conditions.

For the time-course experiment, reactions containing wild-type ZNFX1 were incubated at 37 °C and terminated at the indicated time points by addition of 20 µl of stop buffer containing 0.05% (w/v) SDS, 50 mM EDTA, and 20% (v/v) glycerol. For comparison of RNA unwinding activities, reactions containing wild-type or mutant ZNFX1 proteins were incubated at 37 °C for 30 min before termination with the same stop buffer. Reaction products were resolved by native PAGE gels (90 V, 70 min), imaged using a ChemiDoc MP Imaging System (Bio-Rad), and quantified using ImageJ.

### ATPase activity assay

ATPase activity was measured using the Malachite Green Phosphate Assay Kit (Sigma–Aldrich). Wild-type ZNFX1, the Walker A mutant (K625A), and the blocking helix mutant (BH_Ala) (20 nM each) were incubated in assay buffer containing 20 mM HEPES (pH 7.0), 2 mM MgCl₂, 150 mM KCl, and 1 mM DTT in the presence or absence of 200 nM RNA junction substrate. Reactions were initiated by the addition of ATP to a final concentration of 0.5 mM and incubated at 37 °C for 30 min. The reaction mixtures were diluted threefold, and phosphate production was quantified by measuring absorbance at 620 nm using a Synergy Neo2 microplate reader (BioTek).

### Reporting summary

Further information on research design is available in the Nature Portfolio Reporting Summary linked to this article.

## Supplementary information

Supplementary Information

Reporting Summary

Transparent Peer Review file

## Source data

Source Data

## Data Availability

Plasmids generated in this study are available from the corresponding author upon request. The cryo-EM maps have been deposited in the Electron Microscopy Data Bank (EMDB) under accession codes EMD-75702 (3.71 Å; composite ZNFX1 tetramer), EMD-75741 (3.79 Å; consensus ZNFX1 tetramer), EMD-75718 (3.71 Å; focused-refinement map of one half of the ZNFX1 tetramer), EMD-77053 (3.37 Å; composite ZNFX1–ssRNA–ATPγS dimer complex), EMD-77032 (3.70 Å; consensus ZNFX1–ssRNA–ATPγS dimer complex), and EMD-77051 (3.37 Å; focused-refinement map of one half of the ZNFX1–ssRNA–ATPγS dimer complex). The corresponding atomic coordinates have been deposited in the Protein Data Bank (PDB) under accession codes 11HT https://doi.org/10.2210/pdb11HT/pdb (ZNFX1 tetramer), 11IM https://doi.org/10.2210/pdb11IM/pdb (one half of the ZNFX1 tetramer), 13FZ https://doi.org/10.2210/pdb13FZ/pdb (ZNFX1–ssRNA–ATPγS dimer complex), and 13FU https://doi.org/10.2210/pdb13FU/pdb (one half of the human ZNFX1–ssRNA–ATPγS dimer complex). Previously reported structures used in this study are available from the Protein Data Bank under accession codes 2XZL https://doi.org/10.2210/pdb2XZL/pdb, 2XZO https://doi.org/10.2210/pdb2XZO/pdb, 4B3G https://doi.org/10.2210/pdb4B3G/pdb, and 9QWN https://doi.org/10.2210/pdb9QWN/pdb. Source data are provided in this paper.
