# Supplementary material for: Cryo-EM structures of autoinhibited and RNA-activated ZNFX1 helicase
Source: Nat Commun. 2026 Sep 25;17:11134. doi: 10.1038/s41467-026-78145-0 (PMC13710534; doi:10.1038/s41467-026-78145-0)
Supplement: Supplementary file 1 [file 41467_2026_78145_MOESM1_ESM.pdf]

Supplemental information for

Cryo-EM structures of autoinhibited and RNA-activated ZNFX1 helicase

Qing He, Feng Wang, Feyruz V. Rassool, Stephen B. Baylin, Huilin Li

This document contains

One Supplementary Table

Eleven Supplementary Figures

Supplementary Table 1. Cryo-EM data collection, refinement, and validation statistics

	Autoinhibited ZNFX1 tetramer			ZNFX1 dimer bound to ssRNA and ATPγS		
	Consensus Map (EMD-77032)	Composite Map (EMD-75702) (PDB-11HT)	Local Refinement (EMD-75718) (PDB-11IM)	Consensus Map (EMD-75718)	Composite Map (EMD-77053) (PDB-13FZ)	Local Refinement (EMD-77051) (PDB-13FU)
**Data collection and processing**						
Microscope	FEI Titan Krios					
Magnification	105,000					
Voltage (kV)	300					
Electron exposure (e⁻/Å^2)	50					
Defocus range (μm)	–1.2 to –1.8					
Pixel size (Å/pixel)	0.828					
Symmetry imposed	C2	C1	C1	C2	C1	C1
Initial particle images (no.)	2,232,061			4,186,618		
Final particle images (no.)	129,380	258,760	258,760	131,948	263,896	263,896
Map resolution (Å)	3.79	3.71	3.71	3.70	3.37	3.37
FSC threshold	0.143	0.143	n/a	0.143	0.143	n/a
Map resolution range (Å)	9.0-3.3	9.0-3.2	9.0-3.2	8.0-3.1	6.5-2.9	6.5-2.9
Map sharpening *B* factor (Å^2)	-110.5	-128.3	-128.3	-118.9	-120.3	-120.3
**Refinement**						
Model resolution (Å)	N/A	4.1	4.1	N/A	3.8	3.8
FSC threshold	0.5					
Model resolution range (Å)	N/A	30-3.71	30-3.71	N/A	30-3.37	30-3.37
Model composition						
Non-hydrogen atoms		44040	22020		19220	9610
Protein/RNA residues		5472	2736		2298/26	1149/13
Ligands		4	2		2	1
B factors (Å^2)						
Protein/RNA		106.84	106.82		96.96/94.47	96.36/94.47
Ligand		91.01	92.31		48.52	47.83
R.m.s. deviations						
Bond lengths (Å)		0.005	0.005		0.005	0.005
Bond angles (°)		1.204	1.199		1.141	1.131
Validation						
MolProbity score		2.03	2.02		1.86	1.78
Clash score		9.12	9.50		7.74	7.22
Rotamer outliers (%)		2.13	2.09		1.73	1.44
Ramachandran plot						
Favored (%)		95.82	95.96		96.20	96.16
Allowed (%)		4.18	4.04		3.80	3.84
Disallowed (%)		0.00	0.00		0.00	0.00

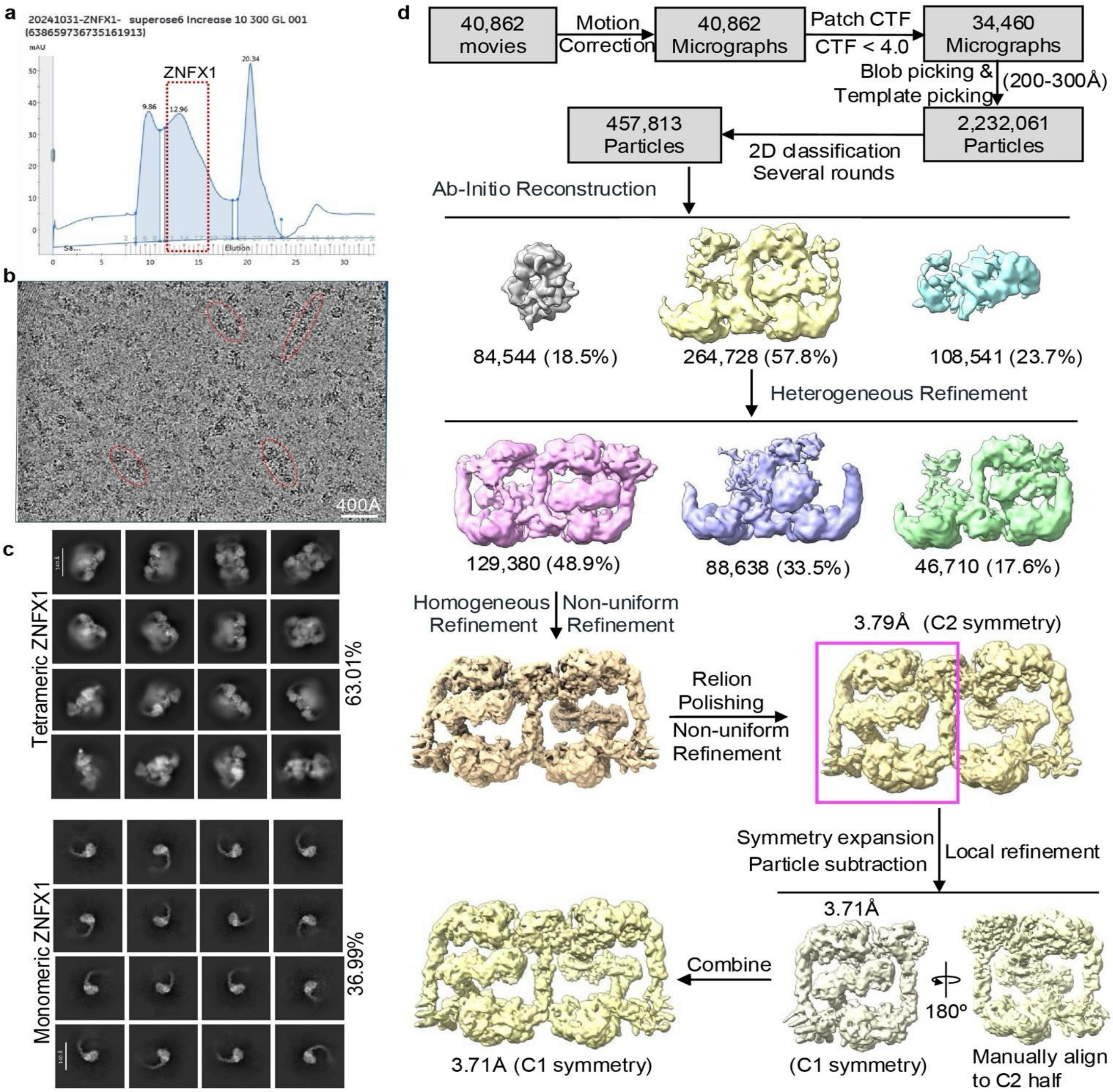

Supplementary Figure 1. Cryo-EM data processing of the autoinhibited apo ZNFX1. a, Size-exclusion chromatography (SEC) profile of purified ZNFX1 expressed in mammalian cells. **b,** Representative cryo-EM micrograph of autoinhibited apo ZNFX1. Short filamentous assemblies are highlighted by red ovals. **c,** Representative 2D class averages of tetrameric and monomeric ZNFX1 particles. The percentages of tetrameric and monomeric particles among the well-defined 2D classes are indicated on the right. The high similarity of the monomer class averages reflects severe preferred orientation. **d**, CryoSPARC (v4.7.1) image-processing workflow for the autoinhibited apo ZNFX1 dataset, leading to the final composite EM map at 3.71 Å resolution.

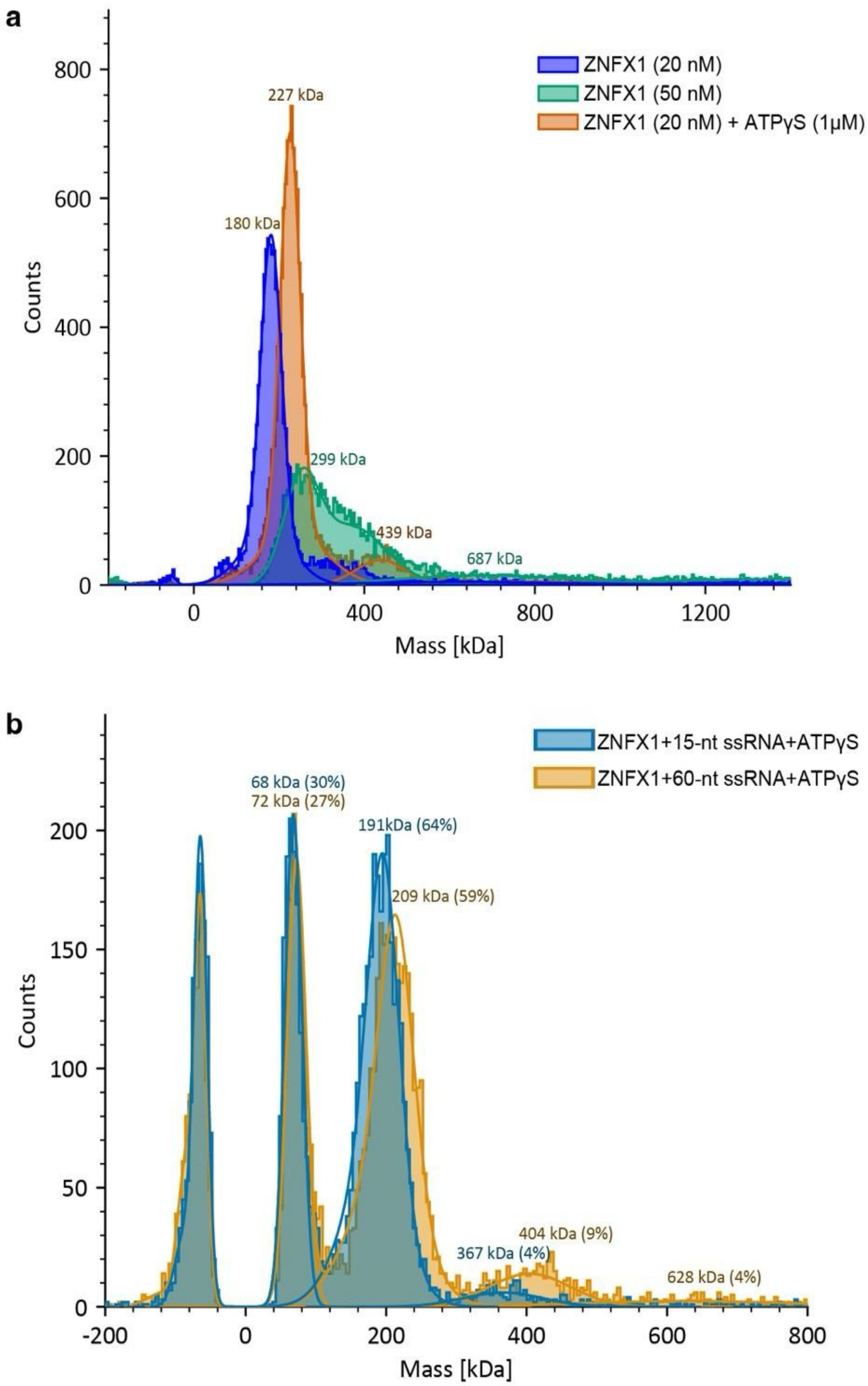

Supplementary Figure 2. Mass photometry analysis of ZNFX1 oligomerization and RNA-induced dimerization. a, Mass photometry profiles of ZNFX1 measured at 20 and 50 nM. Increasing protein concentration broadens the mass distribution, consistent with concentration-dependent self-association. **b**, Mass photometry analysis of ZNFX1 incubated with either 15-nt or 60-nt ssRNA in the presence of ATPγS. Peak molecular masses and the relative abundance of each species are indicated.

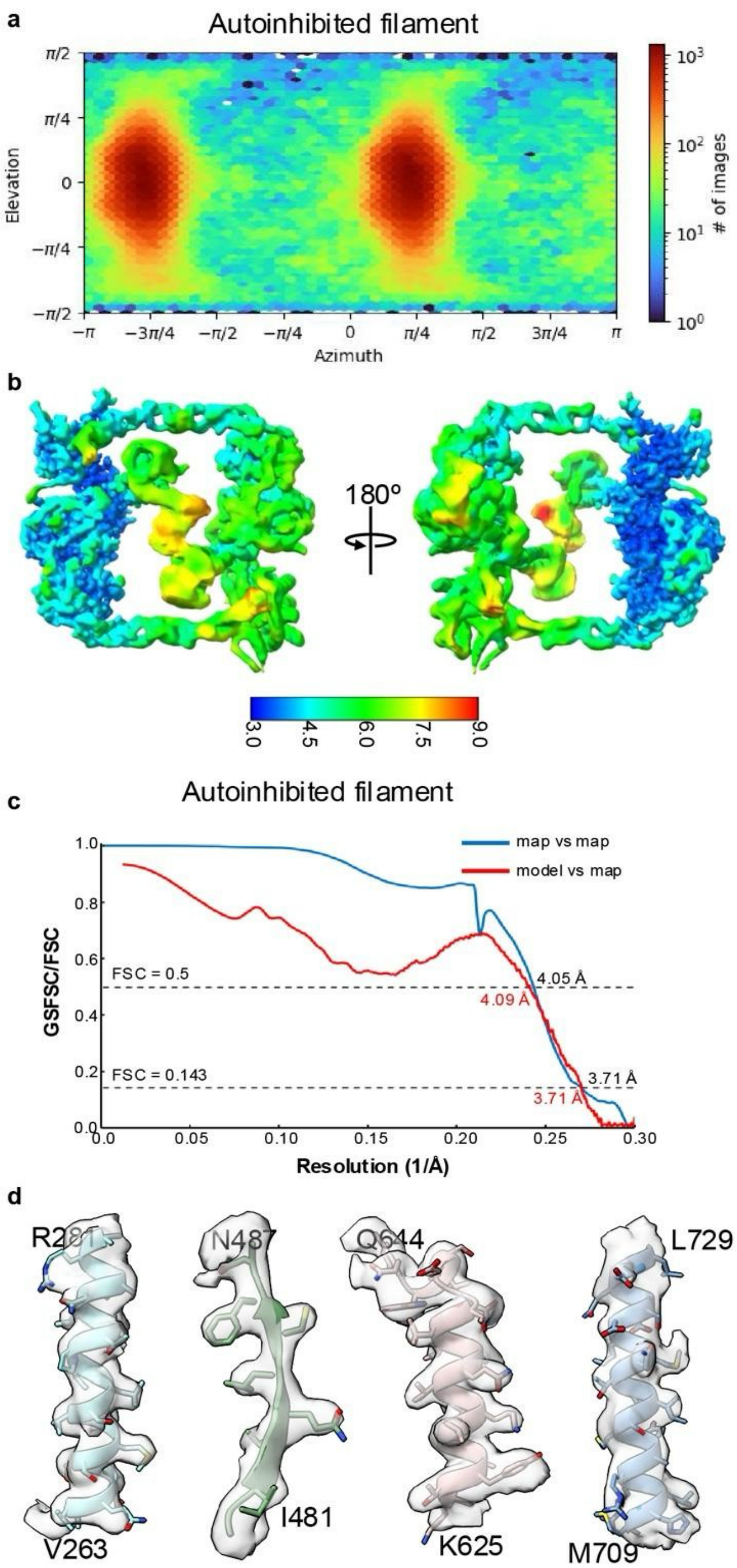

Supplementary Figure 3. Resolution estimation of the focused cryo-EM maps of ZNFX1 in autoinhibited apo and ssRNA-bound states. a, Angular distribution of particle orientations contributing to the final reconstructions of autoinhibited ZNFX1 (left) and the ssRNA-bound complex (right). **b,** Local resolution maps of the autoinhibited (left) and ssRNA-bound (right) maps, color-coded according to resolution. **c**, Gold-standard Fourier shell correlation (FSC) curves for the final cryo-EM maps (blue) and FSC curves between the refined atomic models and the corresponding EM maps (black). **d,** Representative cryo-EM densities from selected regions of autoinhibited (left) and ssRNA-bound (right) structures.

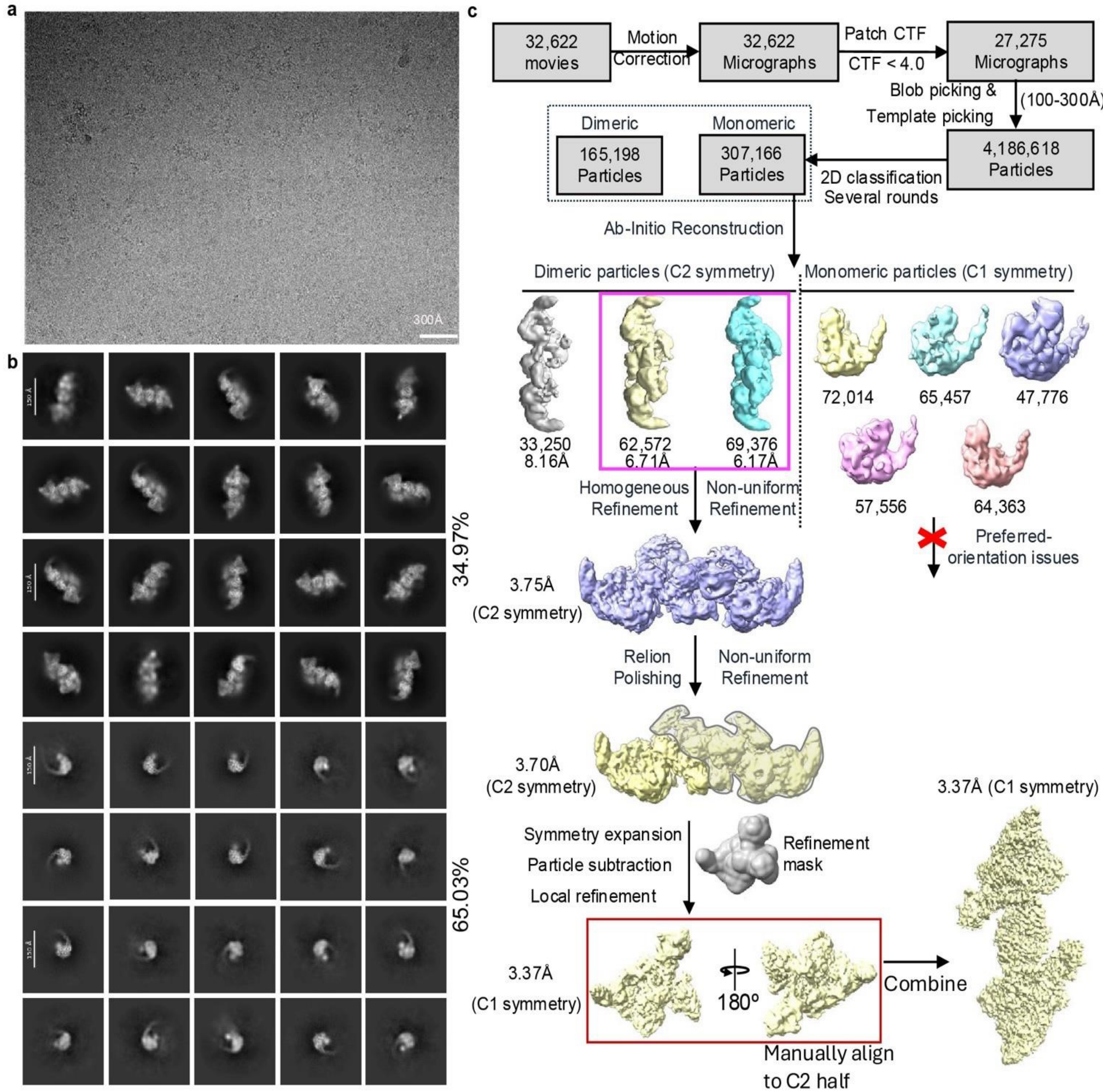

Supplementary Figure 4. Cryo-EM data processing of the ssRNA-bound ZNFX1 complex. a, Representative cryo-EM micrograph of ZNFX1 incubated with 60-nt ssRNA and 1 mM ATPγS. A total of 32,622 micrographs were collected. **b,** Representative 2D class averages of dimeric (top) and monomeric (bottom) ZNFX1 particles. The percentages of dimeric (top) and monomeric (bottom) ZNFX1 particles among the well-defined 2D classes are indicated on the right. The high similarity of the monomeric class averages reflects severe preferred orientation, precluding reliable 3D reconstruction. **c,** CryoSPARC (v4.7.1) image-processing workflow for the ssRNA-bound ZNFX1 dataset, leading to the final composite map at 3.37 Å resolution.

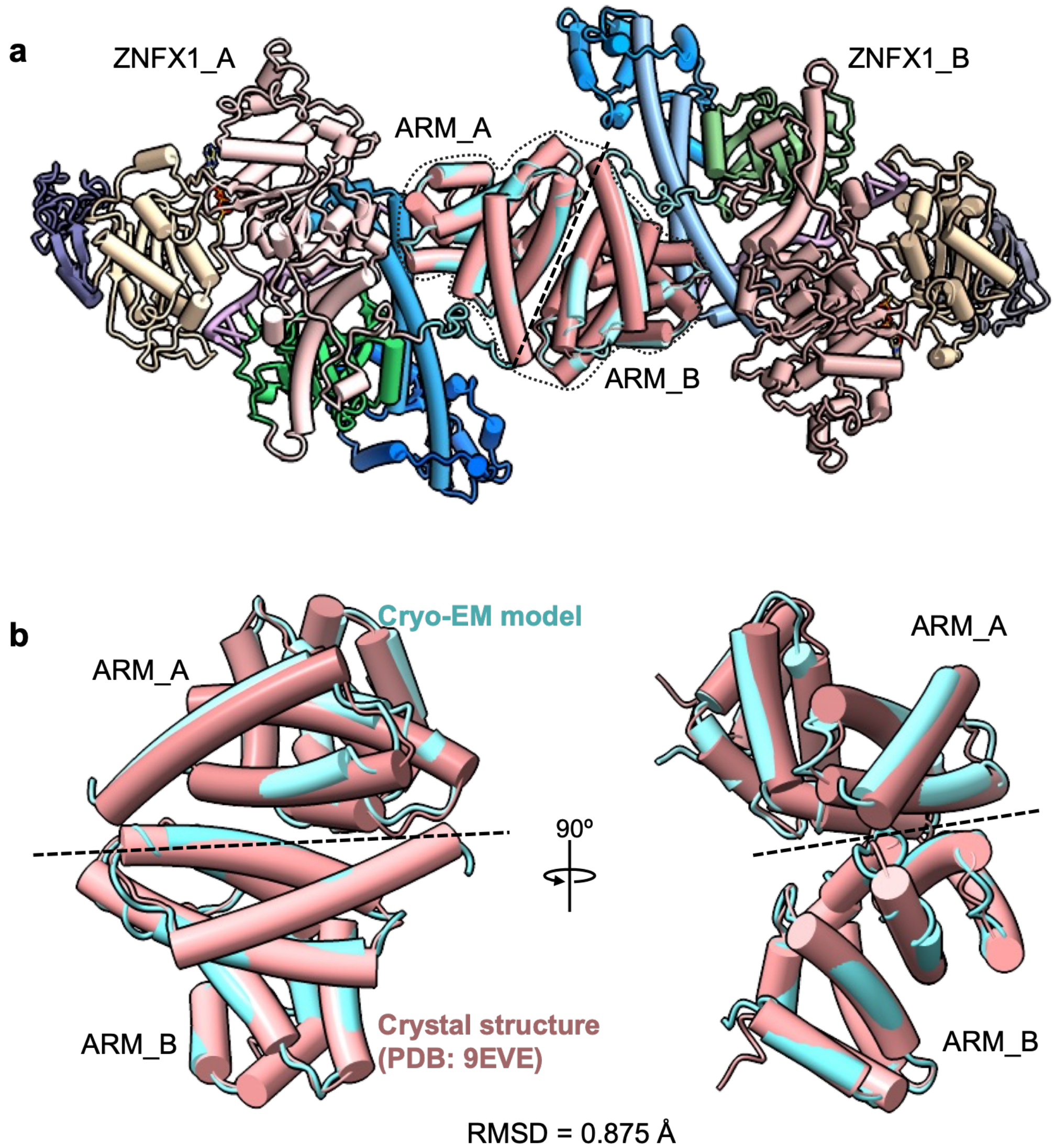

Supplementary Figure 5. Structural comparison of the ARM dimerization mode with the previously reported ARM dimer crystal structure. a, Superposition of the previously reported ARM dimer crystal structure (cyan; PDB 9EVE) onto the ARM domains of the cryo-EM structure of the ZNFX1ssRNA dimer. The cryo-EM model is colored by domains as in Fig. 3b. **b,** Close-up views of the superimposed ARM dimers in two orientations. The two structures align with an RMSD of 0.875 Å over 286 Cα atoms, demonstrating a highly conserved dimerization mode.

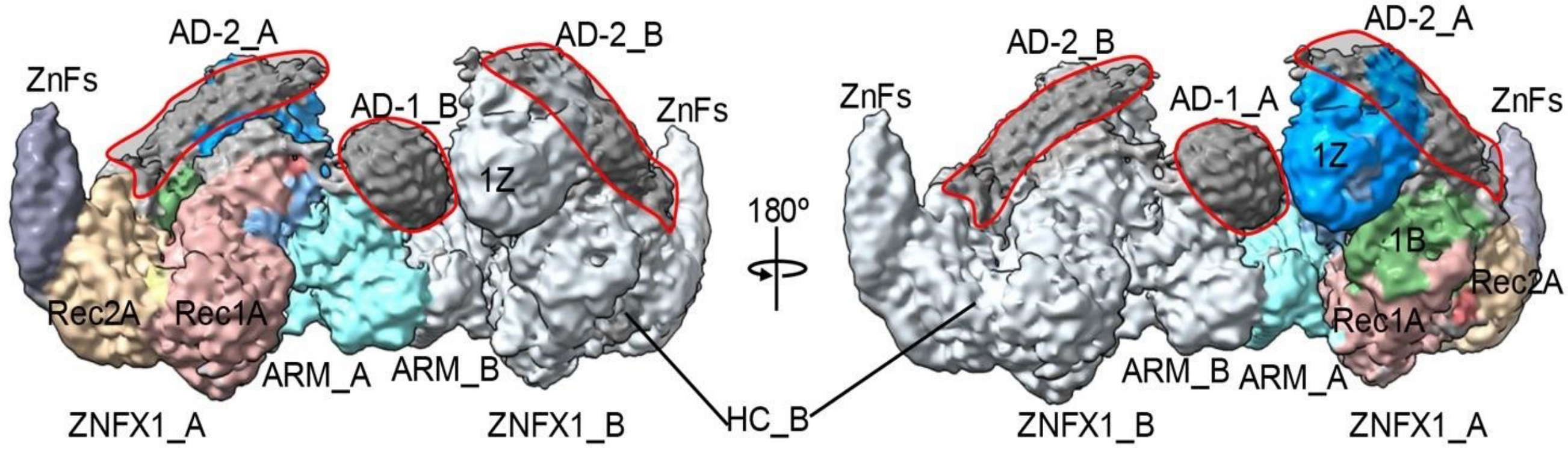

Supplementary Figure 6. Cryo-EM 3D map of the ssRNA-bound ZNFX1 dimer reveals additional unmodeled densities. Front and back views of the cryo-EM 3D reconstruction are shown in surface representation at a low display threshold. One ZNFX1 protomer (A) is colored by domain, whereas the second protomer (B) is shown in gray. Two additional unmodeled densities (AD1 and AD2) are outlined in red and labeled.

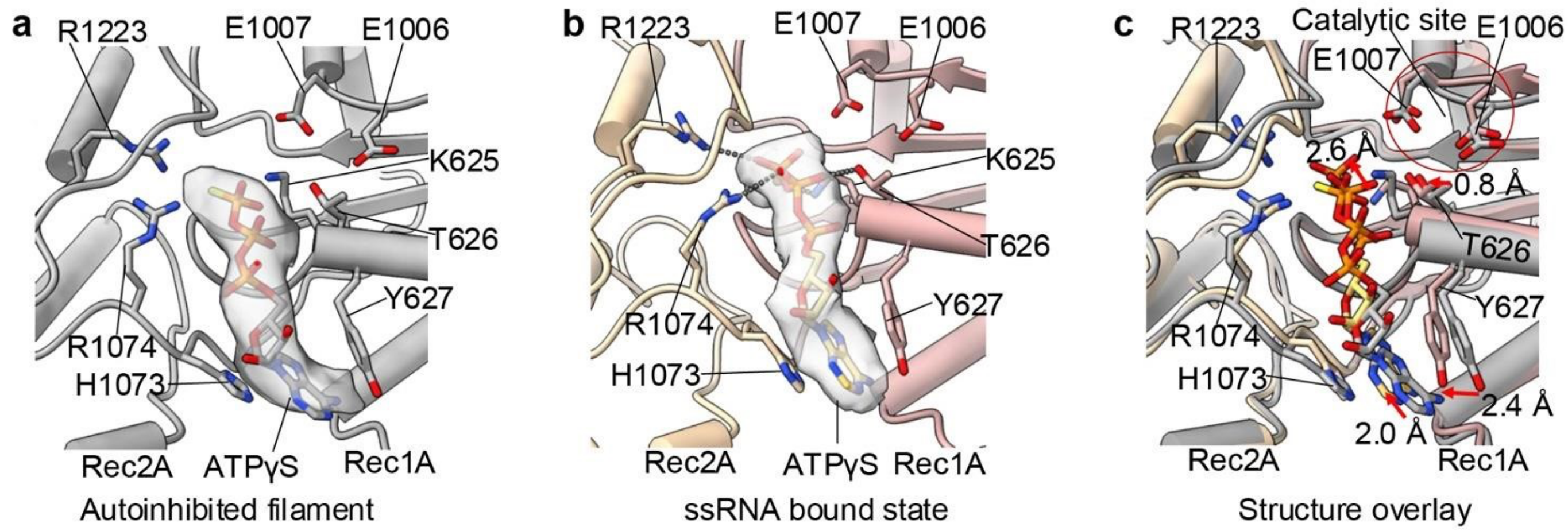

Supplementary Figure 7. Comparison of the ATP-binding site of ZNFX1 in the autoinhibited tetramer and the ssRNA-bound dimer. a-b, Close-up views of the ATP-binding pocket in the autoinhibited (**a**) and ssRNA-bound (**b**) states. ATPγS density is shown as a transparent surface, with interacting residues displayed as sticks and labeled. **c,** Structural superposition of the ATP-binding sites in the autoinhibited and ssRNA-bound states. Red arrows indicate the conformational shifts of ATPγS and surrounding interacting residues between the two states.

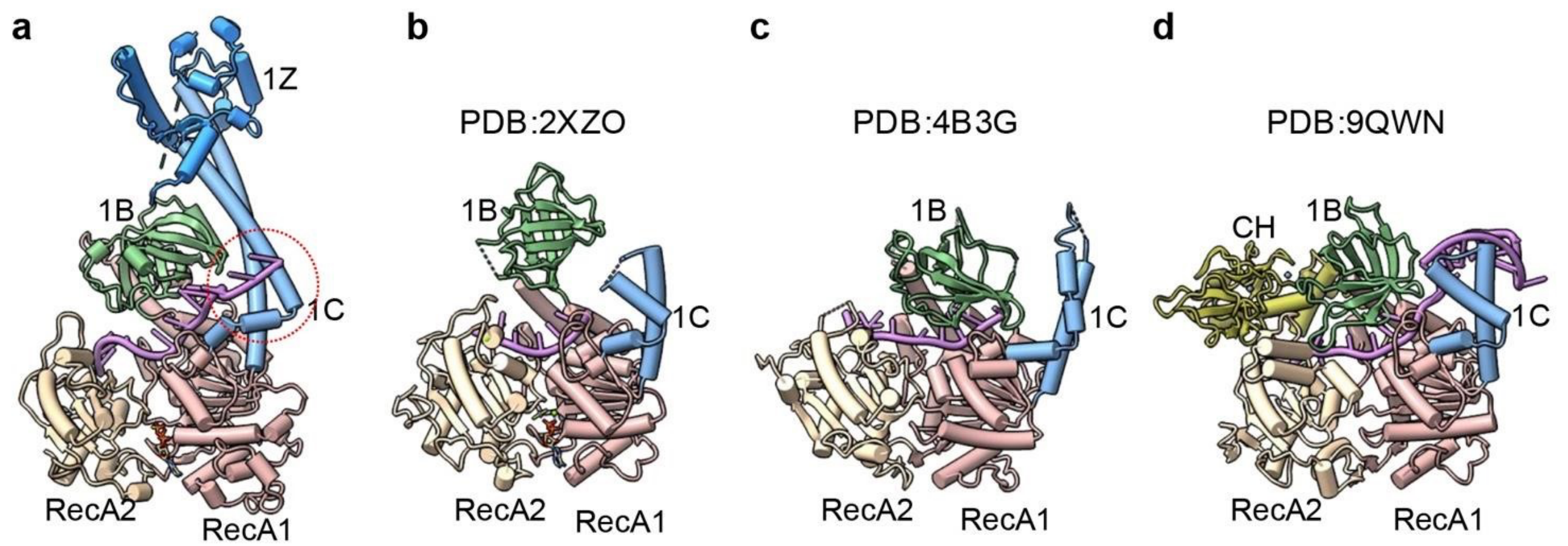

Supplementary Figure 8. Structural comparison of RNA-binding modes in ZNFX1 and other UPF1 helicases. a, Human ZNFX1 bound to a 60-nt ssRNA**. b,** Human UPF1 bound to a 15-nt ssRNA (PDB 2XZO)**. c,** Human IGHMBP2 bound to a 10-nt ssRNA (PDB 4B3G). **d,** Human UPF1 bound to a 15-nt ssRNA (PDB 9QWN). All proteins are shown as cartoon representations in their monomeric forms, with the conserved helicase cores aligned in the same orientation and colored consistently with ZNFX1. The ARM and ZnF domains of ZNFX1 are omitted for clarity. The cysteine–histidine-rich (CH) domain of UPF1 is shown in gold. In ZNFX1, the ssRNA is accommodated by the helicase core and the extended 1C domain, whereas in other UPF1-family helicases the ssRNA passes through the RNA-binding groove formed within the helicase core.

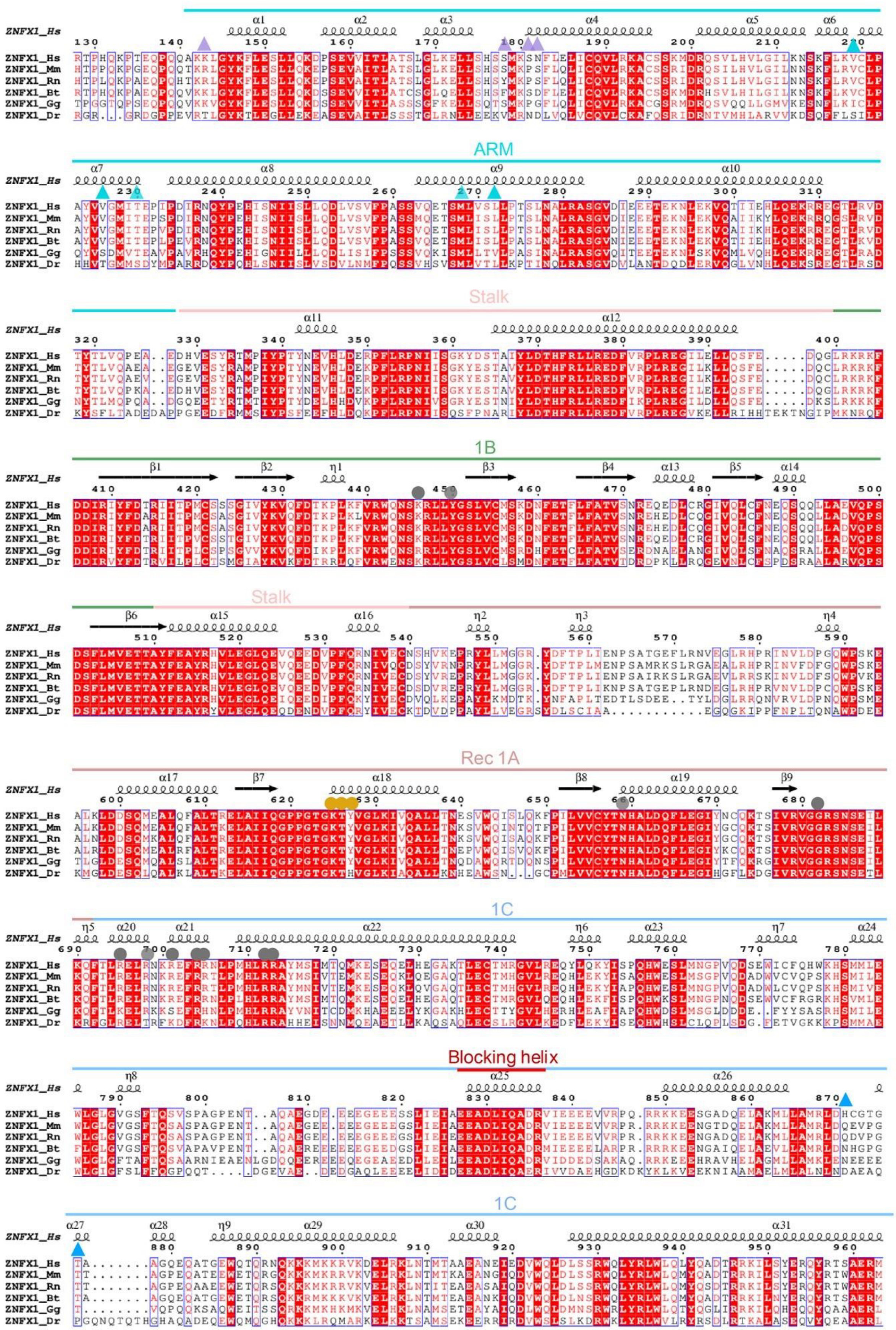

ZNFX1_Hs
ZNFX1_Mm
ZNFX1_Rn
ZNFX1_Bt
ZNFX1_Gg
ZNFX1_Dr
ARM
Stalk
1B
Stalk
Rec 1A
1C
Blocking helix
1C

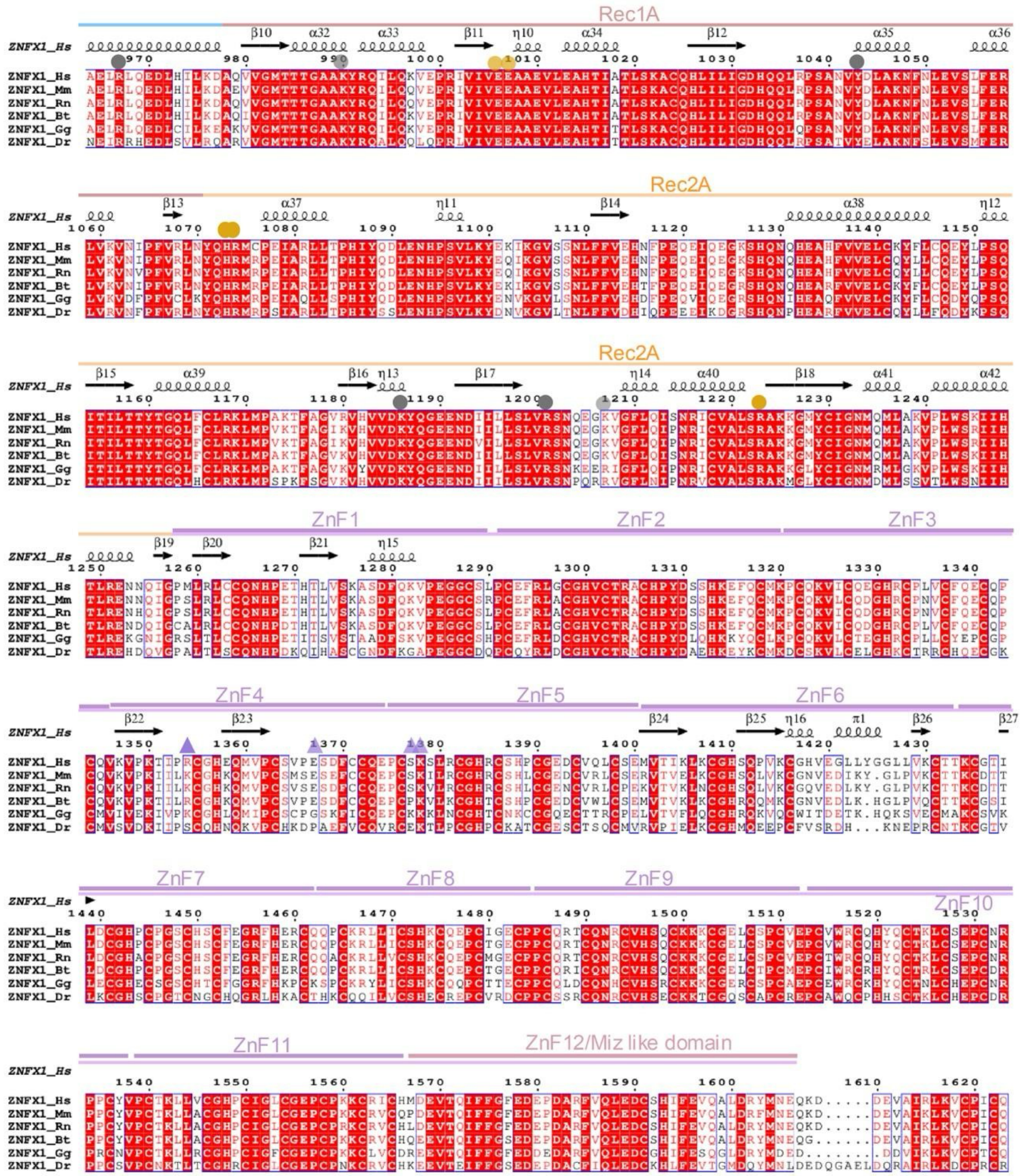

Supplementary Figure 9. Sequence alignment of selected ZNFX1 orthologs highlighting conserved structural and functional features. Sequence alignment of the ARM, helicase core, and ZnF regions from selected ZNFX1 orthologs. Conserved residues are shaded in red. ARM repeats, helicase core subdomains, and individual ZnF motifs (ZnF1–11 and ZnF12/Miz-like domain) are indicated. Residues involved in ATP binding and ssRNA binding are marked by yellow and gray circles, respectively. Residues mediating the ARM–ARM and 1Z–1Z interfaces are indicated by cyan and blue triangles, respectively, whereas residues participating in the ARM–ZnF interface are marked by purple triangles. Species abbreviations: Hs, *Homo sapiens*; Mm, *Mus musculus*; Rn, *Rattus norvegicus*; Bt, *Bos taurus*; Gg, *Gallus gallus;* Dr, *Danio rerio*.

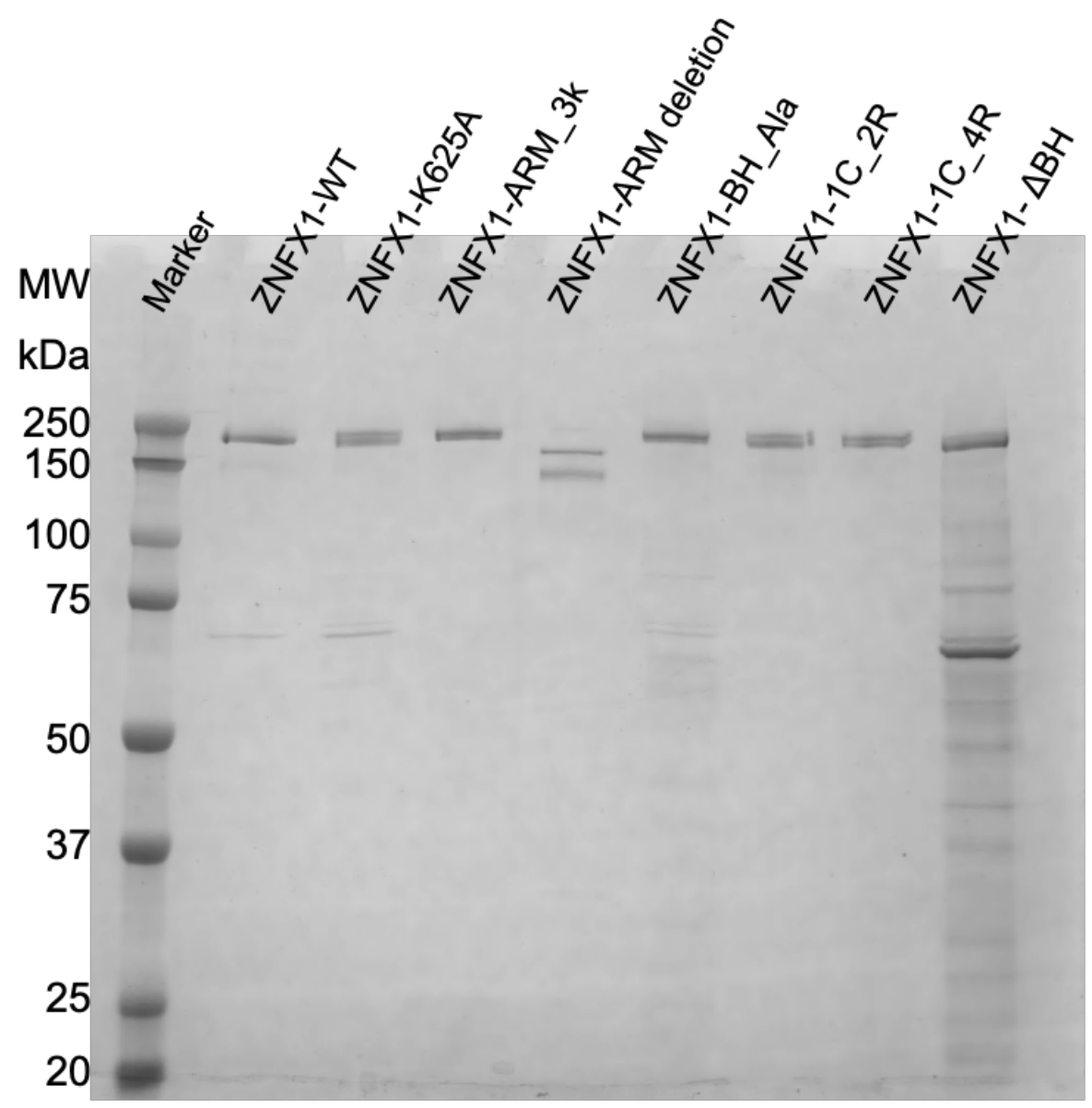

Supplementary Figure 10. Purified wild-type and mutant ZNFX1 proteins. Coomassie-stained SDS–PAGE gel showing purified wild-type (WT) and the indicated mutant proteins used for the biochemical assays. The molecular-weight (MW) markers are shown in the leftmost lane.

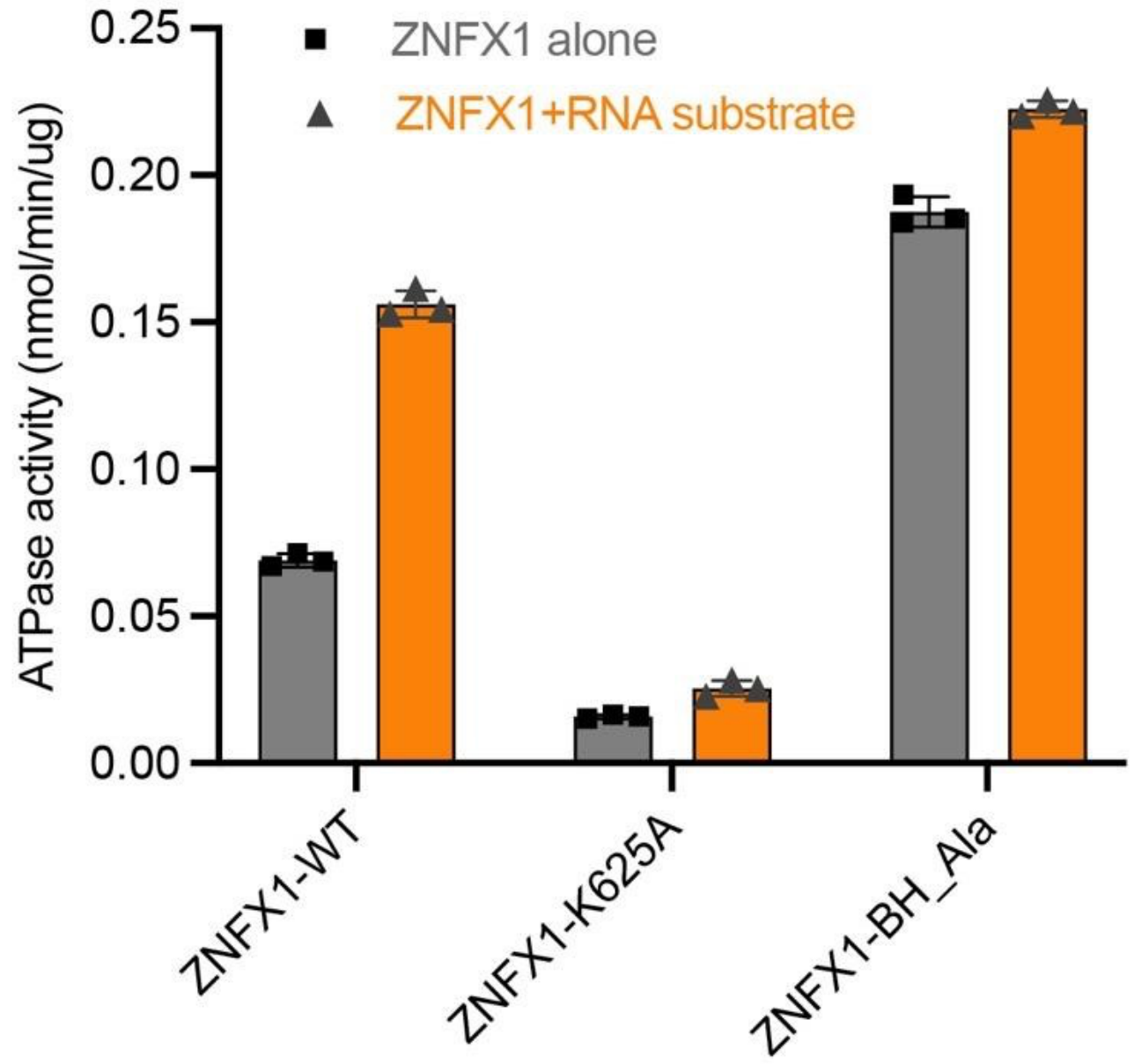

Supplementary Figure 11. ATP hydrolysis activities of wild-type and mutant ZNFX1 proteins. ATPase activities of wild-type (WT), the Walker A mutant (K625A), and the blocking helix mutant (BH_Ala) measured in the absence (gray) and presence (orange) of the RNA junction substrate. ATPase activity was quantified by measuring the release of inorganic phosphate following ATP hydrolysis. Data are presented as mean ± s.d. from three independent experiments. Source data are provided as a Source Data file.
