# Supplementary material for: Cryo-EM structures of autoinhibited and RNA-activated ZNFX1 helicase
Source: Nat Commun. 2026 Sep 25;17:11134. doi: 10.1038/s41467-026-78145-0 (PMC13710534; doi:10.1038/s41467-026-78145-0)
Supplement: Supplementary file 2 [file 41467_2026_78145_MOESM2_ESM.pdf]

nature portfolio

Corresponding author(s): Huilin Li

Last updated by author(s): Sep 2, 2026

Reporting Summary

Nature Portfolio wishes to improve the reproducibility of the work that we publish. This form provides structure for consistency and transparency in reporting. For further information on Nature Portfolio policies, see our Editorial Policies and the Editorial Policy Checklist.

Statistics

For all statistical analyses, confirm that the following items are present in the figure legend, table legend, main text, or Methods section.

n/a	Confirmed	
☐	☒	The exact sample size (*n*) for each experimental group/condition, given as a discrete number and unit of measurement
☒	☐	A statement on whether measurements were taken from distinct samples or whether the same sample was measured repeatedly
☒	☐	The statistical test(s) used AND whether they are one- or two-sided<br>*Only common tests should be described solely by name; describe more complex techniques in the Methods section.*
☒	☐	A description of all covariates tested
☒	☐	A description of any assumptions or corrections, such as tests of normality and adjustment for multiple comparisons
☐	☒	A full description of the statistical parameters including central tendency (e.g. means) or other basic estimates (e.g. regression coefficient) AND variation (e.g. standard deviation) or associated estimates of uncertainty (e.g. confidence intervals)
☒	☐	For null hypothesis testing, the test statistic (e.g. *F*, *t*, *r*) with confidence intervals, effect sizes, degrees of freedom and *P* value noted<br>*Give P values as exact values whenever suitable.*
☒	☐	For Bayesian analysis, information on the choice of priors and Markov chain Monte Carlo settings
☒	☐	For hierarchical and complex designs, identification of the appropriate level for tests and full reporting of outcomes
☒	☐	Estimates of effect sizes (e.g. Cohen's *d*, Pearson's *r*), indicating how they were calculated

Our web collection on statistics for biologists contains articles on many of the points above.

Software and code

Policy information about availability of computer code

Data collection	SerialEM 6.15, BioTek Microplate Readers
Data analysis	Cryo-EM data processing: Cryosparc v 4.7.1<br>Model building and refinement: Phenix v1.21.1-5286, Coot v0.9.8.94, and ChimeraX v 1.8<br>Alpha Screen and RNA unwinding assay: Prism 10<br>ATPase assay: Excel

For manuscripts utilizing custom algorithms or software that are central to the research but not yet described in published literature, software must be made available to editors and reviewers. We strongly encourage code deposition in a community repository (e.g. GitHub). See the Nature Portfolio guidelines for submitting code & software for further information.

Data

Policy information about availability of data

All manuscripts must include a data availability statement. This statement should provide the following information, where applicable:

- Accession codes, unique identifiers, or web links for publicly available datasets
- A description of any restrictions on data availability
- For clinical datasets or third party data, please ensure that the statement adheres to our policy

The cryo-EM maps have been deposited in the Electron Microscopy Data Bank (EMDB) under accession codes EMD-75702 [https://www.ebi.ac.uk/pdbe/entry/

emdb/EMD-75702] (3.71 Å; composite ZNFX1 tetramer), EMD-75741 [https://www.ebi.ac.uk/pdbe/entry/emdb/EMD-75741] (3.79 Å; consensus ZNFX1 tetramer), EMD-75718 [https://www.ebi.ac.uk/pdbe/entry/emdb/EMD-75718] (3.71 Å; focused-refinement map of one half of the ZNFX1 tetramer), EMD-77053 [https://www.ebi.ac.uk/pdbe/entry/emdb/EMD-77053] (3.37 Å; composite ZNFX1–ssRNA–ATPγS dimer complex), EMD-77032 [https://www.ebi.ac.uk/pdbe/entry/emdb/EMD-77032] (3.70 Å; consensus ZNFX1-–ssRNA–ATPγS dimer complex), and EMD-77051 [https://www.ebi.ac.uk/pdbe/entry/emdb/EMD-77051] (3.37 Å; focused-refinement map of one half of the ZNFX1–ssRNA–ATPγS dimer complex). The corresponding atomic coordinates have been deposited in the Protein Data Bank (PDB) under accession codes 11HT [http://doi.org/10.2210/pdb11HT/pdb] (ZNFX1 tetramer), 11IM [http://doi.org/10.2210/pdb11IM/pdb] (one half of the ZNFX1 tetramer), 13FZ [http://doi.org/10.2210/pdb13FZ/pdb] (ZNFX1–ssRNA–ATPγS dimer complex), and 13FU [http://doi.org/10.2210/pdb13FU/pdb] (one half of the human ZNFX1–ssRNA–ATPγS dimer complex). Previously reported structures used in this study are available from the Protein Data Bank under accession codes 2XZL [http://doi.org/10.2210/pdb2XZL/pdb], 2XZO [http://doi.org/10.2210/pdb2XZO/pdb], 4B3G [http://doi.org/10.2210/pdb4B3G/pdb], and 9QWN [http://doi.org/10.2210/pdb9QWN/pdb]. Source data are provided with this paper.

Research involving human participants, their data, or biological material

Policy information about studies with human participants or human data. See also policy information about sex, gender (identity/presentation), and sexual orientation and race, ethnicity and racism.

Reporting on sex and gender	not applicable
Reporting on race, ethnicity, or other socially relevant groupings	not applicable
Population characteristics	not applicable
Recruitment	not applicable
Ethics oversight	not applicable

Note that full information on the approval of the study protocol must also be provided in the manuscript.

Field-specific reporting

Please select the one below that is the best fit for your research. If you are not sure, read the appropriate sections before making your selection.

[x] Life sciences   [ ] Behavioural & social sciences   [ ] Ecological, evolutionary & environmental sciences

For a reference copy of the document with all sections, see nature.com/documents/nr-reporting-summary-flat.pdf

Life sciences study design

All studies must disclose on these points even when the disclosure is negative.

Sample size	For the ZNFX1 tetramer dataset, a single grid collected 40,862 micrographs, from which 2,232,061 particles were initially extracted. After 2D classification, 457,813 particles with clear tetrameric feature contributed to the overall reconstruction, and 129,380 to the final ZNFX1 tetramer structure. For the ssRNA bound ZNFX1 dataset, a single grid collected 32,622 micrographs, which produced 4,186,618 initial particles. After 2D and 3D classification, 131,948 particles were used for the final ZNFX1 dimer bound to ssRNA and ATPγS map. The total dataset size was defined by the amount of microscope access we had, rather than by a prior power analysis or formal estimation of sample size.
Data exclusions	Data were excluded only as part of the standard cryo-EM image-processing workflow. During initial screening, micrographs were discarded if they showed suboptimal CTF estimation, substantial beam-induced motion, outlier defocus values, thick or crystalline ice, or visible contamination. Particles were initially selected using blob-based picking. In later rounds of 2D and 3D classification, particles were grouped according to structural features, and classes corresponding to noise, monomeric ZNFX1, or particles failing to yield well-defined 3D reconstructions were removed. No data were excluded beyond these routine quality-control procedures. All processing and curation steps are detailed in the Methods and illustrated in the Supplementary Figures.
Replication	Protein purification and the ZNFX1-ssRNA-ATPγS complex assembly were repeated multiple times (>5) during method optimization. Final cryo-EM data collection for each structure was collected from a single grid, separately. The alpha screen-RNA binding assay, ATPase assay, and RNA unwinding assay were repeated at least 3 times with the similar results.
Randomization	Randomization was not relevant to this study, which focused on cryo-EM structural characterization and in vitro biochemical assays of purified ZNFX1.
Blinding	Blinding was not applicable to the cryo-EM single-particle analysis. Blinding was also not considered relevant to the biochemical assays, as the identities of the wild-type and mutant proteins were required for sample preparation and experimental setup.

Reporting for specific materials, systems and methods

We require information from authors about some types of materials, experimental systems and methods used in many studies. Here, indicate whether each material, system or method listed is relevant to your study. If you are not sure if a list item applies to your research, read the appropriate section before selecting a response.

Materials & experimental systems

n/a	Involved in the study	
☒	☐	Antibodies
☐	☒	Eukaryotic cell lines
☒	☐	Palaeontology and archaeology
☒	☐	Animals and other organisms
☒	☐	Clinical data
☒	☐	Dual use research of concern
☒	☐	Plants

Methods

n/a	Involved in the study	
☒	☐	ChIP-seq
☒	☐	Flow cytometry
☒	☐	MRI-based neuroimaging

Eukaryotic cell lines

Policy information about cell lines and Sex and Gender in Research

Cell line source(s): The Sf9 cells (GibcoTM Cat# 11496015) and the FreeStyleTM 293-F cells (Catalog R79007)

Authentication: The cell lines were obtained from Thermo Fisher. Expression system was not authenticated in our laboratory.

Mycoplasma contamination: Mycoplasma contamination was not detected.

Commonly misidentified lines (See ICLAC register): No commonly misidentified lines were used.

Plants

Seed stocks: not applicable

Novel plant genotypes: not applicable

Authentication: not applicable
