# Supplementary material for: Cryo-EM structures of autoinhibited and RNA-activated ZNFX1 helicase
Source: Nat Commun. 2026 Sep 25;17:11134. doi: 10.1038/s41467-026-78145-0 (PMC13710534; doi:10.1038/s41467-026-78145-0)
Supplement: Supplementary file 3 [file 41467_2026_78145_MOESM3_ESM.pdf]

nature portfolio

Peer Review File

Cryo-EM structures of autoinhibited and RNA-activated ZNFX1 helicase

Corresponding Author: Professor Huilin Li

This file contains all reviewer reports in order by version, followed by all author rebuttals in order by version.

Version 0:

Reviewer comments:

Reviewer #1

(Remarks to the Author)
ZNFX1 plays a critical role in defense against viral infections. Recently, it has been reported that ZNFX1 functions as an RNA-ubiquitinating E3 ligase, capable of ubiquitinating RNA. RNA acts as an effector that allows ZNFX1 to carry out its function in trans. In the manuscript by He et al., the authors discovered that ZNFX1 forms tetramers, which further assemble into short filaments. They used cryo-EM to reconstruct the structure of the tetrameric complex, which revealed an autoinhibitory mechanism of ZNFX1 in the filamentous arrangement. They further revealed that, upon binding of ssRNA, the filamentous ZNFX1 disassembles and adopt major conformational changes into a dimeric and monomeric form, which activates the ZNFX1. These findings provide a structural explanation for the transition from autoinhibitory filamentous state to activated ssRNA-bound state. The manuscript describes the structural mechanism of the ZNFX1's activation process in good detail and is timely given the importance of the protein's role in innate immunity. The following points need to be addressed before the publication.

1.While the tetramerization interface is proposed, the critical residues involved in the interaction between ZNFX1 subunits need to be further analyzed. For instance, how conserved are these interfacial residues? Is there any mutagenesis experiment to validate the formation of tetramer's critical roles in autoinhibition?

2.Have the authors compared the ZNFX1 dimer-RNA structure with the ZNFX1 monomer-RNA structure to determine whether the RNA binding sites are the same? Such a comparison would better illustrate the RNA binding characteristics of the dimer. It is critical to clarify whether the RNA in the dimer-RNA structure represents a single contiguous RNA molecule, rather than segments from two separate RNAs.

3.What is the maximum length of the filament? Is it always composed of three tetramers? Furthermore, the authors may discuss the potential physiological significance of the filamentous assembly in cells.

4.In Line 155-156, the authors mentioned the blocking helix as a helix enriched of positive charged residues. But in the supplementary Figure 8, the helix appears to contains mostly negatively charged residues.

5.Lines 290-292 suggest that ZNFX1 binds RNA in a sequence-independent manner and associates with various RNA species. What biochemical evidence supports this? How does ZNFX1 specifically recognize viral RNA? In the final section, the predicted ARM domain of ZNFX1 has a positively charged surface that may bind dsRNA. Does it also bind ssRNA? This is particularly relevant given that reference 20 reported minimal dsRNA binding by ZNFX1 in fluorescence polarization measurements （ref 20 Figure 2C. Additionally, the model in Figure 4F would benefit from experimental validation, such as binding or helicase assays.

6.In line 221-228, regarding the weak density attributed to the E3 module, how was this assignment confirmed? It is difficult to distinguish the additional density in 2D class averages and no comparison of the 2D averages is shown.

7.In Figure 1, would it be possible to also color-code the domains of the other three monomers within the tetramer on the short filament? Using distinct colors would make the figure clearer and easier to understand immediately, preventing the need for readers to refer back to it after viewing the next figure. For clarity, the colored domains in Figures 2 and 3 could be labeled with their names (e.g., in a legend or directly on the figure).

8.The images in the article are unclear and become blurry when enlarged. There is a potential citation error in line 136: the reference (20) may be incorrect and should likely be (26).

Reviewer #2

(Remarks to the Author)
In this manuscript, He et al determine two cryo-EM structures of the innate immune helicase ZNFX1. One structure is of a filamentous form in the absence of nucleic acid while another structure reveals an RNA-bound dimer structure. The authors discuss the potential functional relevance of these structures and compare them to other UPF1-family helicases.
ZNFX1 is a highly unusual helicase/E3 ligase which makes any insight into its structure and function of considerable interest. Overall, the structures are of relatively good quality, as much as can be expected from their ~3.7 A resolution, enabling modelling of sidechains and molecular interactions in well resolved parts and docking of alphafold models in parts at lower local resolution. While the structures and their analysis is generally good, the manuscript offers almost no functional data. Thus, all of the conclusions are highly speculative. Testing some of the main speculations would substantially improve the manuscript. Otherwise, I would suggest toning down the speculation and focusing more on the structural data present.
Major points
It is stated that filaments are an inhibitory conformation which are broken up by RNA to activate the enzyme as a main finding in the abstract. However, this is not shown. With structural data alone the causality cannot be determined. It is also not clear which activity of the enzyme is being referred to and this should be specified. I assume they are referring to RNA binding. In this case, the model would be that RNA binding relieves inhibition of RNA binding, which requires some more explanation. If they mean ubiquitination activity, then this is very speculative without any ubiquitination assays as how the E3 activity of ZNFX1 is regulated is currently much less well understood than how nucleic acids binds a UPF1-family helicase.
Specific points:

- Does mutating the blocking helix affect the affinity for RNA or RNA stimulated ATPase activity

- The authors should demonstrate with biochemical/biophysical assays whether filaments can bind RNA or if RNA can disassemble filaments. There are numerous ways the latter can be demonstrated. The cryoEM data provides some insight into this but it should be clarified in the methods whether the same protein preparation was used for both datasets. Furthermore, it would be helpful to include the ratio of monomer to oligomer particles for both datasets (i.e. next to the 2D classes). Regardless, variation in ice quality etc. between grids can strongly affect such interpretation.

- The mass photometry would potentially provide insight into these issues but is currently ambiguous – in the size range shown, monomers, dimers and tetramers should be clearly distinguishable but instead there seems to just be a broad peak with low signal which is centered on the predicted mass of a dimer. The authors should attempt these measurements with RNA present. But perhaps another technique would give clearer results – for example analytical SEC at different concentrations with or without RNA.

- As there is no structure of the monomer with and without RNA it is difficult to determine what effects are caused by the filament or by the RNA. For example, the authors makes a claim that in the dimer the 1Z domain is moved away from the helicase pulling out the blocking helix (line 259). But the 1Z domain is flexible in the dimer so should not constrain this helix. Indeed, in the preprint from Naydenova et al, they determine a monomer ZNFX1 structure without RNA and see the blocking helix bound.

- As the authors have tried to link structural state to E3 ligase activity, the manuscript could be considerably strengthened by performing ubiquitin ligase assays, Does the RNA used in the structure activate the E3 activity of ZNFX1? Can they isolate filaments from SEC and compare their E3 activity with monomers?

- The authors use a chapter in the results section and most of Fig. 4 to discuss an Alphafold3 model of the ARM domain bound to dsRNA, even claiming it provides a model for dsRNA separation. This is speculation, not data, and should be entirely removed unless data are provided. Specifically, the dsRNA binding ability of the isolated ARM domain should be determined with and without mutating the predicted interface. Then, the binding of structured substrates to full-length ZNFX1 as well as the helicase activity of ZNFX1 should be determined with and without mutating the interface. Alternatively, they can determine an experimental structure. If they do include the AF3 model (with the above supporting data), confidence metrics should be provided as well as showing what happens if the full length monomer and dimer are predicted in AF3 with dsRNA or 5'overhang ss/dsRNA.

- The authors state that filament formation is cooperative but this has not been shown and only short filaments are present in the cryoEM data.

Minor points

- The authors say they can determine the register of the RNA sequence – it would be useful to have a figure showing the RNA density.

- Figure 3C is not very clear – perhaps the authors could find a better way to represent this.

- It might be useful to compare the ARM dimerization mode to the previously published ARM dimer crystal structure.

- Line 155 – presumably negatively charged, not positive.

Reviewer #3

(Remarks to the Author)
Summary
This manuscript presents cryo-EM structures of full-length human ZNFX1 in two states: an autoinhibited tetrameric/filamentous form and an ssRNA-bound dimer. The authors argue that ZNFX1 is held inactive through concentration-dependent filament assembly, in which a blocking helix occludes the RNA-binding groove, and that ssRNA binding breaks apart these assemblies and remodels the helicase core into an active conformation. The filament-based autoinhibition mechanism via a blocking helix is new and distinguishes ZNFX1 from other UPF1-family helicases, which use intramolecular autoinhibition. The RNA-driven switch from filament to active dimer is relevant to understanding how ZNFX1 coordinates its helicase and E3 ligase activities (Grabarczyk et al. 2025). The structures sit alongside other examples of filament-based regulation in innate immune sensors (RIG-I, MDA5, cGAS). A concurrent preprint (Naydenova et al.) reports ZNFX1 filament structures and an RNA-bound monomer; this study extends that work by defining the tetramer as the repeating unit and presenting the dimeric RNA-bound state. The structures are well described and the domain-level analysis is thorough. However, there is a significant concern with the cryo-EM processing methodology, a complete absence of biochemical validation for the proposed mechanisms, and an unsupported unwinding model that need to be addressed.

Major Concerns
Major Point 1.
The data processing workflow for the autoinhibited tetramer (Supplementary Figure 1D) contains a methodological error in the symmetry expansion and local refinement steps. After C2 symmetry expansion, every particle orientation is duplicated with the C2 operator applied, placing both monomers into a common reference frame within a single expanded stack. The authors correctly perform signal subtraction of the other asymmetric unit prior to local refinement, which is appropriate for improving mask performance. However, this makes the subsequent step harder to explain: two separate masks are applied to the two halves of the dimer, producing two maps at different resolutions (3.71 Å and 3.64 Å). The two different resolutions obtained from what should be an equivalent procedure strongly suggest that either the expansion was not performed correctly, or that focused refinement was carried out on the non-expanded stack and mislabelled. The correct procedure is to refine with one mask on the asymmetric unit from the expanded and subtracted stack, producing one map, which is then docked into a lower-resolution map of the full particle to generate the composite tetramer. The authors need to clarify and correct this workflow.

Major Point 2.
The blocking helix is proposed to mediate autoinhibition by occluding the RNA-binding groove, but no mutagenesis is presented to test this. Deletion or charge-reversal mutations of the blocking helix, with effects on RNA binding or oligomeric state measured, would be needed to support this claim, have the author's made any attempts to make mutants to test these claims?

Major Point 3.
The ARM–ARM interface is identified as the primary dimerisation contact and the backbone of filament propagation, but no interface-disrupting mutations are tested. Similarly, the 1Z–1Z and ARM–ZnF interfaces are described in detail and proposed to stabilise the tetramer and filament, yet none of these interactions are validated biochemical studies. Without at least key point mutations at these interfaces, the functional importance of these contacts remains assertion rather than demonstration. Are there any in vivo data to support this claim?

Major Point 4.
While ARM–dsRNA binding has been observed in a cellular context (Wang et al. 2019, ref 12), the specific binding mode and residues proposed here (K143, K147, K180) rest entirely on an AlphaFold3 prediction. No in vitro binding data are provided in this study to validate the predicted binding interface or confirm direct, reconstituted ARM–dsRNA interaction, given the lower resolution of the map. Since this predicted interface forms the basis of the unwinding model in Figure 4F, the gap between cell-based observation and the structural claims made here should be addressed experimentally.

Major Point 5.
The proposed unwinding mechanism (Figure 4F) lacks experimental support. It depends on the unvalidated dsRNA–ARM interaction (Major Point 4), an assumed "pulling force" generated by ssRNA translocation, and analogy to the Sen1 helicase. No unwinding assays are presented and there is no evidence that ZNFX1 can simultaneously engage ssRNA through the helicase core and dsRNA through the ARM domain. Presenting this as a concluding mechanistic figure overstates the data as it has been presented. This should either be supported with biochemistry, removed or and discussed with appropriate caveats.

Major Point 6.
Both datasets have preferred orientation problems. Monomeric particles in both the apo and ssRNA-bound datasets had orientation bias. For the tetramer and dimer reconstructions, the angular distribution plots (Supplementary Figure 3A) are shown but not discussed in the main text. The authors should comment on whether orientation anisotropy affects map quality

in specific regions or any of their claims.

Minor Concerns
Minor Point 1.
The main text reports ZNFX1 concentrations as “~20 nM” and then “0.02 to 0.2 μM” in the same passage, while Supplementary Figure 2 uses nM throughout. Units should be consistent.

Minor Point 2.
The Figure 1F caption describes an “atomic model” of a single ZNFX1 subunit, but the text states that ZnF1–6 was modelled by fitting an AlphaFold prediction into lower-resolution density. The caption should distinguish which regions are experimentally built and which are docked AlphaFold models, as the current wording is misleading for a non-specialist reader.

Minor Point 3.
The comparison between the ZNFX1 blocking helix and the UPF1 RNA-binding site (Figures 1E–F) would be more effective as a direct structural superposition rather than two separate panels. Aligning the helicase cores would immediately show that the blocking helix sits in the equivalent position to ssRNA in UPF1.

Minor Point 4.
For the ssRNA-bound dimer, C2 symmetry was imposed and local refinement used a soft-edged mask. It is unclear whether symmetry expansion was also applied here. Given the issues with the tetramer workflow (Major Point 1), the authors should clarify the exact processing steps for the dimer reconstruction.

Minor Point 5.
The Methods section states a defocus range of “–1.2 to –1.0 μm” while Supplementary Table 1 reports “–1.2 to –1.8 μm.” These should be reconciled.

Minor Point 6.
The filament architecture is described as “reminiscent of a double-stranded arrangement.” This could be misread as referring to double-stranded nucleic acid rather than a double-helical filament. Consider rewording to “double-helical arrangement” or similar.

Version 1:

Reviewer comments:

Reviewer #1

(Remarks to the Author)
My concerns are adequately addressed. I recommend to accept this work now.

Reviewer #2

(Remarks to the Author)
The authors have responded appropriately to the reviewer feedback, improving their paper by adding biochemical experiments to support their claims, focusing their conclusions on their data and increasing the detail of the methods sections. I now support publication of this manuscript in Nature Communications.

Reviewer #3

(Remarks to the Author)
I thank the authors for a thorough revision. The added biochemistry, comprising ATPase assays, AlphaScreen binding assays, and the first direct demonstration of ATP-dependent RNA unwinding by purified human ZNFX1, addresses my central concern, and the cryo-EM processing has been corrected as requested.

On my previous points: Major Point 1 (symmetry expansion and local refinement) is fully resolved, and the revised workflow, particle counts and map resolutions are internally consistent. Major Points 4 and 5 are substantively addressed. Major Point 3 is not tested experimentally, but the model has been restructured so that it no longer depends on those interfaces, which I accept. Major Point 6 is now discussed in the text as I asked, though a quantitative anisotropy metric would still be preferable. All six minor points are fixed. I have two remaining concerns.

1. Modelled contacts are presented as observed contacts. The Methods state that the 1Z-1Z interface was built from an AlphaFold prediction because the density was insufficient, and the Results that the zinc-finger region was fitted into lower-resolution density. That disclosure is not carried through to the claims: both interfaces are then described in the same register as the ARM-ARM interface, with individual contacts named as though observed, marked as interface residues in the alignment, and used for a conservation contrast in the Discussion. Of the three, only the ARM-ARM interface is well resolved and independently corroborated by the published ARM dimer crystal structure. Please identify the predicted contacts as such wherever they are asserted, and temper the conservation argument accordingly.

2. The functional dissection needs more cautious framing. Deleting the ARM domain and mutating its RNA-binding surface each reduce unwinding only modestly, and indistinguishably from one another and from the less extensively substituted 1C variant; only the Walker A and most extensively substituted 1C mutants impair it severely. This indicates a contributory rather than obligatory role for the ARM with this short junction substrate, so the stated requirement for engagement by both the ARM and the helicase core overstates the data; "progressively" is likewise unsupported for the 1C series. The two ARM constructs also appear to be assigned their values in opposite order in the text and the figure key.

Open Access This Peer Review File is licensed under a Creative Commons Attribution 4.0 International License, which permits use, sharing, adaptation, distribution and reproduction in any medium or format, as long as you give appropriate credit to the original author(s) and the source, provide a link to the Creative Commons license, and indicate if changes were made.
In cases where reviewers are anonymous, credit should be given to 'Anonymous Referee' and the source.
The images or other third party material in this Peer Review File are included in the article’s Creative Commons license, unless indicated otherwise in a credit line to the material. If material is not included in the article’s Creative Commons license and your intended use is not permitted by statutory regulation or exceeds the permitted use, you will need to obtain permission directly from the copyright holder.
To view a copy of this license, visit https://creativecommons.org/licenses/by/4.0/

Point-by-point responses to the reviewers' comments

Reviewer #1 (Remarks to the Author)

ZNFX1 plays a critical role in defense against viral infections. Recently, it has been reported that ZNFX1 functions as an RNA-ubiquitinating E3 ligase, capable of ubiquitinating RNA. RNA acts as an effector that allows ZNFX1 to carry out its function in trans. In the manuscript by He et al., the authors discovered that ZNFX1 forms tetramers, which further assemble into short filaments. They used cryo-EM to reconstruct the structure of the tetrameric complex, which revealed an autoinhibitory mechanism of ZNFX1 in the filamentous arrangement. They further revealed that, upon binding of ssRNA, the filamentous ZNFX1 disassembles and adopt major conformational changes into a dimeric and monomeric form, which activates the ZNFX1. These findings provide a structural explanation for the transition from autoinhibitory filamentous state to activated ssRNA-bound state. The manuscript describes the structural mechanism of the ZNFX1's activation process in good detail and is timely given the importance of the protein's role in innate immunity. The following points need to be addressed before the publication.

Author response: We thank the reviewer for the positive assessment of our work and for the constructive comments. To address the reviewer's concerns and strengthen the mechanistic conclusions of the study, we generated a series of ZNFX1 mutant proteins and performed additional biochemical assays. These new experiments provide functional validation of our structural findings and have been incorporated into the revised manuscript.

1. While the tetramerization interface is proposed, the critical residues involved in the interaction between ZNFX1 subunits need to be further analyzed. For instance, how conserved are these interfacial residues? Is there any mutagenesis experiment to validate the formation of tetramer's critical roles in autoinhibition?

Author response: We thank the reviewer for this helpful suggestion. We first examined the conservation of residues involved in the three tetramer interfaces. Sequence alignment showed that many residues mediating the ARM-ARM and 1Z-1Z interactions are well conserved across species, whereas residues comprising the ARM–ZnF interface exhibit more limited conservation (revised **Supplementary Fig. 9**).

We also investigated the role of oligomerization in autoinhibition through functional analysis of a blocking-helix mutant. Our new biochemical data indicates that oligomerization is not strictly required for autoinhibition. Accordingly, we have revised the manuscript to moderate our previous interpretation. We now propose that both monomeric and tetrameric/filamentous ZNFX1 adopt the same autoinhibited conformation mediated by the

blocking helix, whereas filament formation is concentration dependent and likely represents a storage form that stabilizes autoinhibited ZNFX1 molecules rather than the sole autoinhibited state.

2. Have the authors compared the ZNFX1 dimer-RNA structure with the ZNFX1 monomer-RNA structure to determine whether the RNA binding sites are the same? Such a comparison would better illustrate the RNA binding characteristics of the dimer. It is critical to clarify whether the RNA in the dimer-RNA structure represents a single contiguous RNA molecule, rather than segments from two separate RNAs.

Author response: We thank the reviewer for this insightful suggestion. Unfortunately, the experimental structure of monomeric ZNFX1 bound to ssRNA structure has not been released in the Protein Data Bank, precluding a direct structural comparison of the RNA-binding sites in monomeric and dimeric ZNFX1.

To further investigate the relationship between RNA length and ZNFX1 oligomerization, we performed new mass photometry analyses in the presence of either a 15-nt or a 60-nt ssRNA. Under both conditions, ZNFX1 existed as monomers and dimers. However, the proportion of dimeric species increased substantially in the presence of the 60-nt ssRNA (revised **Supplementary Fig. 2b**), indicating that longer ssRNA promoting ZNFX1 dimerization more efficiently.

Regarding the RNA observed in the cryo-EM structure, the RNA density associated with each protomer is discontinuous, and therefore, we cannot determine whether it represents a single continuous RNA molecule bridging the two ZNFX1 protomers or two independently bound RNA molecules. Nevertheless, the increased abundance of dimers in the presence of the 60-nt ssRNA is consistent with the possibility that a longer RNA can simultaneously engage both ZNFX1 molecules within a dimer. This interpretation is also supported by the recent study of Grabarczyk et al (Cell, 2025), which showed that long ssRNA substrates promote ZNFX1 multimerization. We have incorporated this discussion into the revised manuscript.

3. What is the maximum length of the filament? Is it always composed of three tetramers? Furthermore, the authors may discuss the potential physiological significance of the filamentous assembly in cells.

Author response: We thank the reviewer for this helpful suggestion. The filaments observed in our cryo-EM micrographs are approximately 500–1000 Å in length, corresponding to about 2–4 tetrameric repeats. Thus, the filaments are not always composed of three tetramers but exhibit variable lengths.

We have expanded the Discussion to address the potential physiological significance of filament formation. Based on our structural and biochemical data, together with the recent study by Naydenova et al (bioRxiv, 2025), which reported even longer ZNFX1 filaments and higher-order helical bundles at elevated protein concentrations, we propose that filament formation is concentration dependent and likely represents a storage form of autoinhibited ZNFX1. Upon RNA binding, these assemblies are remodeled into RNA-bound monomeric and dimeric species that are competent for helicase activity.

4. In Line 155-156, the authors mentioned the blocking helix as a helix enriched of positive charged residues. But in the supplementary Figure 8, the helix appears to contain mostly negatively charged residues.

Author response: We thank the reviewer for catching this error. The blocking helix is enriched in negatively charged residues that mimics an RNA substate. We have corrected the text accordingly.

5. Lines 290-292 suggest that ZNFX1 binds RNA in a sequence-independent manner and associates with various RNA species. What biochemical evidence supports this? How does ZNFX1 specifically recognize viral RNA? In the final section, the predicted ARM domain of ZNFX1 has a positively charged surface that may bind dsRNA. Does it also bind ssRNA? This is particularly relevant given that reference 20 reported minimal dsRNA binding by ZNFX1 in fluorescence polarization measurements（ref 20 Figure 2C). Additionally, the model in Figure 4F would benefit from experimental validation, such as binding or helicase assays.

Author response: We thank the reviewer for these insightful suggestions. Previous studies have shown that ZNFX1 associates with both single-stranded and double-stranded nucleic acids *in vitro* and *in vivo* (Wang et al., Nature cell biology, 2019; Stojanovic et al., Cancer Research, 2025; Grabarczyk et al., Cell, 2025). We have revised the manuscript to better summarize these findings and to clarify that the molecular basis for viral RNA specificity remains an important question for future investigation.

To experimentally validate the predicted RNA-binding surface of the ARM domain, we performed AlphaScreen RNA-binding assays using the purified ARM domain. We found that the ARM domain binds both dsRNA and ssRNA, with higher affinity for ssRNA. In contrast, the ARM-3K mutant, in which Lys143, Lys147, and Lys180 were replaced with glutamate residues, showed no detectable binding to either dsRNA or ssRNA (revised **Fig. 5b**), demonstrating that these positive charged residues are critical for RNA binding.

To further test the proposed unwinding mechanism, we performed RNA helicase assays using wild-type and mutant ZNFX1 proteins. These experiments demonstrate, for the first time, that ZNFX1 possesses ATP-dependent RNA unwinding activity and that efficient unwinding requires both ATP hydrolysis and RNA binding by the ARM domain (**Fig. 5e**).

Together, these new biochemical data provide functional support for the proposed RNA-binding model and the mechanistic interpretation derived from our structural analyses.

6. In line 221-228, regarding the weak density attributed to the E3 module, how was this assignment confirmed? It is difficult to distinguish the additional density in 2D class averages, and no comparison of the 2D averages is shown.

Author response: We thank the reviewer for raising this important point. To clarify the assignment of the additional density, we have revised **Fig. 3a** by relabeling the major structural features in representative 2D class averages, including the helicase core, the ZnF region, and the ARM-ARM interface. The additional density cannot be assigned to any of the well-resolved domains of ZNFX1. Because the C-terminal E3 ligase module is the only major region that remains unresolved in our cryo-EM structure, we tentatively attributed this density to the E3 module. We have revised both the text and figure legend to emphasize the tentative nature of this assignment.

7. In Figure 1, would it be possible to also color-code the domains of the other three monomers within the tetramer on the short filament? Using distinct colors would make the figure clearer and easier to understand immediately, preventing the need for readers to refer back to it after viewing the next figure. For clarity, the colored domains in Figures 2 and 3 could be labeled with their names (e.g., in a legend or directly on the figure).

Author response: We thanks the reviewer for these helpful suggestions. We have revised **Figures 1-3** to improve their clarity by color-coding the domains of all four ZNFX1 protomers within the tetramer and by adding domain labels directly to the figures where appropriate. These revisions make the structural organization and domain assignments easier to follow throughout the manuscript.

8. The images in the article are unclear and become blurry when enlarged. There is a potential citation error in line 136: the reference (20) may be incorrect and should likely be (26).

Author response: We thank the reviewer for pointing out these issues. We have replaced the affected figures with higher resolution versions to improve image quality and corrected the reference citation in the revised manuscript.

Reviewer #2 (Remarks to the Author):

In this manuscript, He et al determine two cryo-EM structures of the innate immune helicase ZNFX1. One structure is of a filamentous form in the absence of nucleic acid while another structure reveals an RNA-bound dimer structure. The authors discuss the potential functional relevance of these structures and compare them to other UPF1-family helicases. ZNFX1 is a highly unusual helicase/E3 ligase which makes any insight into its structure and

function of considerable interest. Overall, the structures are of relatively good quality, as much as can be expected from their ~3.7 A resolution, enabling modelling of sidechains and molecular interactions in well resolved parts and docking of AlphaFold models in parts at lower local resolution. While the structures and their analysis is generally good, the manuscript offers almost no functional data. Thus, all of the conclusions are highly speculative. Testing some of the main speculations would substantially improve the manuscript. Otherwise, I would suggest toning down the speculation and focusing more on the structural data present.

Author response: We thank the reviewer for the thoughtful and constructive comments, as well as for the positive assessment of our structural analyses. To address the primary concern regarding the lack of functional validation, we generated a series of ZNFX1 mutant proteins and performed additional biochemical analyses, including ATPase assays, the AlphaScreen RNA-binding assays, and the RNA helicase unwinding assays using both wild-type and mutant ZNFX1 proteins. These new experiments provide functional validation of the proposed RNA-binding mode and substantially strength the mechanistic conclusions derived from our structural studies. We have also revised the manuscript throughout to moderate speculative interpretations where appropriate.

Major points

It is stated that filaments are an inhibitory conformation which are broken up by RNA to activate the enzyme as a main finding in the abstract. However, this is not shown. With structural data alone the causality cannot be determined. It is also not clear which activity of the enzyme is being referred to, and this should be specified. I assume they are referring to RNA binding. In this case, the model would be that RNA binding relieves inhibition of RNA binding, which requires some more explanation. If they mean ubiquitination activity, then this is very speculative without any ubiquitination assays as how the E3 activity of ZNFX1 is regulated is currently much less well understood than how nucleic acids binds a UPF1-family helicase.

Author response: We thank the reviewer for this important comment. We agree that our original wording overstated the causal relationship between filament disassembly and ZNFX1 activation. Accordingly, we have revised the manuscript to clarify that our study focuses on the structural basis of RNA binding and RNA helicase, rather than the regulation of the E3 ligase activity. The C-terminal E3 ligase module was not resolved in our cryo-EM maps. Although several recent studies have shown that RNA binding stimulates the E3 ligase activity of ZNFX1 (Naydenova et al. bioRxiv, 2025; Grabarczyk et al. Cell, 2025; Squair et al. Molecular Cell 2026), elucidating the structural basis of this activity will require stabilization of the E3 module and is beyond the scope of the present study.

To strengthen the mechanistic conclusions, we performed new biochemical experiments, including ATPase, AlphaScreen RNA-binding, and RNA helicase unwinding assays. These

experiments provide direct functional support for the proposed RNA-binding mode and RNA helicase mechanism.

In addition, we have revised our interpretation of the filamentous assemblies. Our cryo-EM analyses indicate that ZNFX1 alone exists predominantly as monomers, whereas tetramers and short filaments represent a concentration-dependent population. Together with the new biochemical data, we now propose that both monomeric and filamentous ZNFX1 adopt the same blocking-helix-mediated autoinhibited conformation. In this model, ssRNA competes with the blocking helix for the RNA-binding channel, thereby promoting conformational remodeling and disassembly of higher-order assemblies, rather than demonstrating a direct causal relationship between filament disassembly and enzymatic activation.

Specific points:

- Does mutating the blocking helix affect the affinity for RNA or RNA stimulated ATPase activity

Author response: We thank the reviewer for this helpful suggestion. To investigate the role of the blocking helix, we generated two ZNFX1 mutants, a blocking-helix deletion mutant (ZNFX1-ΔBH) and a blocking helix alanine-substitution mutant (ZNFX1-BH_Ala). Although ZNFX1-ΔBH formed aggregates and could not be purified to homogeneity, ZNFX1-BH_Ala was purified successfully and used for biochemical analyses.

We found that ZNFX1-BH_Ala reduced RNA unwinding helicase activity compared with wild-type ZNFX1 (revised **Fig. 5e**). In contrast, in the absence of RNA, the mutant displayed higher basal ATPase activity than the wild-type ZNFX1. Addition of RNA stimulated ATPase activity by approximately 2.2-fold for wild-type and only 1.2-fold for ZNFX1-BH_Ala, (revised **Supplementary Fig. 11**). These results indicate that the blocking helix suppresses basal ATPase activity in the absence of RNA and contribute to efficient coupling of ATP hydrolysis to RNA unwinding.

We did not directly measure the RNA-binding affinity of the BH_Ala mutant; however, the ATPase and RNA-unwinding assays demonstrate that the blocking helix plays an important role in regulating helicase function.

- The authors should demonstrate with biochemical/biophysical assays whether filaments can bind RNA or if RNA can disassemble filaments. There are numerous ways the latter can be demonstrated. The cryoEM data provides some insight into this, but it should be clarified in the methods whether the same protein preparation was used for both datasets. Furthermore, it would be helpful to include the ratio of monomer to oligomer particles for both datasets (i.e. next to the 2D classes). Regardless, variation in ice quality etc. between grids can strongly affect such interpretation.

Author response: We thank the reviewer for this excellent suggestion. To further evaluate the effect of RNA on ZNFX1 oligomerization, we reanalyzed our cryo-EM datasets and performed additional mass photometry experiments.

First, we have clarified in the Methods section that the same preparation of purified ZNFX1 protein was used for both the apo and ssRNA-bound cryo-EM datasets, with the latter prepared by incubating the protein with a 60-nt ssRNA substrate.

Second, we quantified the relative abundance of the different particle populations and now present these values alongside the representative 2D class averages. In the apo dataset, monomers accounted for ~37% of the well-defined particles, whereas tetramers/filamentous particles accounted for ~63%, and no dimers were observed (revised Supplementary Fig. 1). In contrast, in the ssRNA-bound dataset, monomers and dimers represented ~65% and ~35% of the particles, respectively, whereas tetrameric/filamentous assemblies were no longer detected (revised **Supplementary Fig. 4**). These observations indicate that addition of the 60-nt ssRNA is accompanied by loss of the higher-order assemblies.

Finally, we performed additional mass photometry analyses in the presence of either a 15-nt or a 60-nt ssRNA. ZNFX1 remained predominantly monomeric with the 15-bt ssRNA, whereas the proportion of dimeric species increased in the presence of the longer 60-nt ssRNA (revised **Supplementary Fig. 2b**). Together, these independent cryo-EM and mass photometry analyses support the conclusion that longer ssRNA promotes remodeling of the higher-order ZNFX1 assemblies into monomeric and dimeric species.

- The mass photometry would potentially provide insight into these issues but is currently ambiguous – in the size range shown, monomers, dimers and tetramers should be clearly distinguishable but instead there seems to just be a broad peak with low signal which is centered on the predicted mass of a dimer. The authors should attempt these measurements with RNA present. But perhaps another technique would give clearer results – for example analytical SEC at different concentrations with or without RNA.

Author response: We thank the reviewer for this helpful suggestion. We agree that the original mass photometry measurement collected at higher concentration were difficult to interpret because of the very broad mass distribution. Accordingly, we have removed the 200 nM dataset from the revised **Supplementary Fig. 2a**. The broad distribution observed at 50 nM is consistent with concentration-dependent formation of ZNFX1 filaments of heterogenous lengths, in agreement with the recent report by Naydenova et al. (bioRxiv, 2025).

To address the reviewer's suggestion directly, we performed new ZNFX1 mass photometry analyses in the presence of ssRNA substrates (**Supplementary Fig. 2b**). In the presence of a 15-nt ssRNA, ZNFX1 remained predominantly monomeric, with only a small population of dimeric species. In contrast, the proportion of dimeric species increased substantially in the presence of a 60-nt ssRNA. These results demonstrate that the RNA length influences the oligomeric state of ZNFX1 and are fully consistent with our cryo-EM observation.

Because the new mass photometry experiments provided a clear and independent assessment of RNA-dependent changes in ZNFX1 oligomerization, we did not pursue additional analytical size-exclusion chromatography experiments.

- As there is no structure of the monomer with and without RNA it is difficult to determine what effects are caused by the filament or by the RNA. For example, the authors make a claim that in the dimer the 1Z domain is moved away from the helicase pulling out the blocking helix (line 259). But the 1Z domain is flexible in the dimer so should not constrain this helix. Indeed, in the preprint from Naydenova et al, they determine a monomer ZNFX1 structure without RNA and see the blocking helix bound.

Author response: We thank the reviewer for this insightful comment and agree with the concern. Based on our reanalysis of the structural data together with the recent study by Naydenova et al., we now conclude that apo ZNFX1 exists as a mixture of autoinhibited monomers and tetramers/filaments, with the relative abundance of each species depending on protein concentration. We therefore propose that the tetramers and filaments represent a concentration-dependent storage form of autoinhibited ZNFX1 rather than a distinct autoinhibited state.

Accordingly, we have revised the manuscript to remove our previous statement that movement of the 1Z domain displaces the blocking helix from the RNA-binding groove. Instead, we now describe the repositioning of the 1Z domain and the displacement of the blocking helix as structural changes associated with RNA binding, without implying a direct causal relationship between these events.

- As the authors have tried to link structural state to E3 ligase activity, the manuscript could be considerably strengthened by performing ubiquitin ligase assays, Does the RNA used in the structure activate the E3 activity of ZNFX1? Can they isolate filaments from SEC and compare their E3 activity with monomers?

Author response: We thank the reviewer for this important suggestion. We agree that ubiquitination assays would be valuable for defining the relationship between the structural states and its E3 ligase activity. However, because the C-terminal E3 ligase module was not resolved in our cryo-EM maps, we have revised the manuscript to clarify that the present

study focuses on structural basis of RNA binding and RNA helicase activity rather than the regulation of the E3 ligase activity.

Recent studies have demonstrated that RNA binding stimulates E3 ligase activity of ZNFX1 (Naydenova et al., bioRxiv, 2025; Grabarczyk et al., Cell, 2025; Squair et al., Molecular Cell, 2026). Nevertheless, the structural basis of this activation remains unknown. We therefore believe that mechanistic investigation of the E3 ligase activity will require stabilization of the flexible E3 module and is beyond the scope of the present study. We have revised the manuscript accordingly.

- The authors use a chapter in the results section and most of Fig. 4 to discuss an Alphafold3 model of the ARM domain bound to dsRNA, even claiming it provides a model for dsRNA separation. This is speculation, not data, and should be entirely removed unless data are provided. Specifically, the dsRNA binding ability of the isolated ARM domain should be determined with and without mutating the predicted interface. Then, the binding of structured substrates to full-length ZNFX1 as well as the helicase activity of ZNFX1 should be determined with and without mutating the interface. Alternatively, they can determine an experimental structure. If they do include the AF3 model (with the above supporting data), confidence metrics should be provided as well as showing what happens if the full length monomer and dimer are predicted in AF3 with dsRNA or 5'overhang ss/dsRNA.

Author response: We thank the reviewer for this excellent suggestion. To experimentally validate the AlphaFold3-predicted ARM-RNA interface, we performed a series of new biochemical experiments.

First, we purified the isolated ARM domain and an RNA binding mutant (ARM-3K), in which the three predicted RNA-binding residues (Lys143, Lys147, and Lys180) were substituted by glutamate residues. AlphaScreen assay showed that wild-type ARM domain binds both dsRNA and ssRNA, with higher affinity for ssRNA, whereas the ARM-3K mutant showed no detectable binding to either substrate (**Fig. 5b**). These results provide direct experimental validation of the predicted ARM-RNA interface.

Second, we performed RNA helicase assays using full-length wild-type and a panel of mutant proteins with an RNA substrate containing a 15-bp dsRNA duplex and an 18-nt 5'-ssRNA overhang. Both the ARM-3K mutant and the ARM-deletion mutant exhibited reduced RNA unwinding activity compared to wild-type ZNFX1 (**Fig. 5e**), demonstrating that ARM-mediated RNA binding contributes to efficient RNA unwinding.

Finally, to strengthen the structural modeling, we used AlphaFold3 to predict a ZNFX1 monomer, containing the ARM domain and helicase core, bound to an ss/dsRNA junction substrate and included the corresponding confidence metrics (pTM and ipTM scores) for the predicted models in the revised figures. Together, these new structural predictions and

biochemical data provide functional support for the proposed RNA-binding model and RNA-unwinding mechanism.

- The authors state that filament formation is cooperative, but this has not been shown, and only short filaments are present in the cryoEM data.

Author response: We thank the reviewer for this important comment and agree that our original wording overstated the available evidence. We have removed the term "cooperative" from the revised manuscript because our cryo-EM data don't directly demonstrate cooperative filament assembly.

We have also added an analysis of filament length, showing that the filaments observed in our cryo-EM micrographs are approximately 500–1000 Å in length, corresponding to about 2–4 tetrameric repeats. Based on the tetramer structure, we propose that these short filaments could be extended through the conserved terminal interfaces. Consistent with this interpretation, Naydenova et al (bioRxiv, 2025) reported longer ZNFX1 filaments and higher order helical bundles at higher concentrations than those used in our study. Thus, we now interpret our structures as representing short, concentration-dependent filament assemblies, rather than evidence for cooperative filament formation.

Minor points

- The authors say they can determine the register of the RNA sequence – it would be useful to have a figure showing the RNA density.

Author response: We thank the reviewer for this helpful suggestion. We have added a new panel (revised **Fig. 4b**) showing the cryo-EM density of the bound ssRNA together with the fitted atomic model, illustrating the RNA register and the quality of the density.

- Figure 3C is not very clear – perhaps the authors could find a better way to represent this.

Author response: We thank the reviewer for this helpful suggestion. We have revised **Fig. 3c** to improve its clarity by relabeling the key structural elements, outlining the relatively stable ARM and 1C domains, and adding curved red arrows to highlight the rigid-body-like domain movements induced by RNA binding.

- It might be useful to compare the ARM dimerization mode to the previously published ARM dimer crystal structure.

Author response: We thank the reviewer for this helpful suggestion. We have added a structural superposition of the ARM–ARM dimer observed in our cryo-EM structure with the previously reported ARM dimer crystal structure in the revised **Supplementary Fig. 5**. The

comparison demonstrates that the two structures adopt essentially the same dimerization mode.

- Line 155 – presumably negatively charged, not positive.

Author response: We thank the reviewer for catching this error. The text has been corrected to indicate that the blocking helix is enriched in negatively charged residues.

Reviewer #3 (Remarks to the Author)

Summary. This manuscript presents cryo-EM structures of full-length human ZNFX1 in two states: an autoinhibited tetrameric/filamentous form and an ssRNA-bound dimer. The authors argue that ZNFX1 is held inactive through concentration-dependent filament assembly, in which a blocking helix occludes the RNA-binding groove, and that ssRNA binding breaks apart these assemblies and remodels the helicase core into an active conformation. The filament-based autoinhibition mechanism via a blocking helix is new and distinguishes ZNFX1 from other UPF1-family helicases, which use intramolecular autoinhibition. The RNA-driven switch from filament to active dimer is relevant to understanding how ZNFX1 coordinates its helicase and E3 ligase activities (Grabarczyk et al. 2025). The structures sit alongside other examples of filament-based regulation in innate immune sensors (RIG-I, MDA5, cGAS). A concurrent preprint (Naydenova et al.) reports ZNFX1 filament structures and an RNA-bound monomer; this study extends that work by defining the tetramer as the repeating unit and presenting the dimeric RNA-bound state. The structures are well described, and the domain-level analysis is thorough. However, there is a significant concern with the cryo-EM processing methodology, a complete absence of biochemical validation for the proposed mechanisms, and an unsupported unwinding model that need to be addressed.

Author response: We thank the reviewer for the thoughtful and constructive comments, as well as for the positive assessment of our structural analyses. To address the reviewer's major concerns, we have revised the cryo-EM data processing workflow, performed additional biochemical analyses, including ATPase, AlphaScreen RNA-binding, and RNA helicase unwinding assays using wild-type and mutant ZNFX1 proteins, and revised the mechanistic model accordingly. We believe that the new data substantially strength the manuscript and address the reviewer's concerns.

Major Concerns

Major Point 1. The data processing workflow for the autoinhibited tetramer (**Supplementary Fig. 1d**) contains a methodological error in the symmetry expansion and local refinement steps. After C2 symmetry expansion, every particle orientation is duplicated with the C2 operator applied, placing both monomers into a common reference frame within a single expanded stack. The authors correctly perform signal subtraction of the other

asymmetric unit prior to local refinement, which is appropriate for improving mask performance. However, this makes the subsequent step harder to explain: two separate masks are applied to the two halves of the dimer, producing two maps at different resolutions (3.71 Å and 3.64 Å). The two different resolutions obtained from what should be an equivalent procedure strongly suggest that either the expansion was not performed correctly, or that focused refinement was carried out on the non-expanded stack and mislabeled. The correct procedure is to refine with one mask on the asymmetric unit from the expanded and subtracted stack, producing one map, which is then docked into a lower-resolution map of the full particle to generate the composite tetramer. The authors need to clarify and correct this workflow.

Author response: We thank the reviewer for identifying this important issue. We agree with the reviewer's assessment and have revised the cryo-EM data process workflow accordingly. Specifically, we repeated the symmetry expansion, signal subtraction, and local refinement following the procedure recommended by the reviewer and re-refined the cryo-EM maps. The revised workflow also resulted in modest improvements in the final reconstructions, as reported in the revised manuscript. We have updated the Methods section and the corresponding data-processing workflows and reconstruction figures to accurately reflect the revised procedure.

Major Point 2. The blocking helix is proposed to mediate autoinhibition by occluding the RNA-binding groove, but no mutagenesis is presented to test this. Deletion or charge-reversal mutations of the blocking helix, with effects on RNA binding or oligomeric state measured, would be needed to support this claim, have the authors made any attempts to make mutants to test these claims?

Author response: We thank the reviewer for this helpful suggestion. To directly test the role of the blocking helix, we generated two ZNFX1 mutant constructs, a blocking-helix deletion mutant (ZNFX1-ΔBH) and a blocking helix alanine-substitution mutant (ZNFX1-BH_Ala). Although ZNFX1-ΔBH formed aggregates and could not be purified to homogeneity, ZNFX1-BH_Ala was purified successfully and subjected to biochemical characterization.

ZNFX1-BH_Ala exhibited reduced RNA unwinding activity compared with wild-type ZNFX1 (revised **Fig. 5e**). In contrast, in the absence of RNA, the mutant displayed higher basal ATPase activity than wild-type ZNFX1, whereas RNA stimulated ATPase activity by approximately 2.2-fold for the wild-type protein but only 1.2-fold for the ZNFX1-BH_Ala, (revised **Supplementary Fig. 11**). These findings indicate that the blocking helix suppresses basal ATPase activity in the absence of RNA while contributing to efficient coupling of ATP hydrolysis to RNA unwinding, thereby providing functional support for its role in ZNFX1 autoinhibition.

Major Point 3. The ARM–ARM interface is identified as the primary dimerisation contact and the backbone of filament propagation, but no interface-disrupting mutations are tested. Similarly, the 1Z–1Z and ARM–ZnF interfaces are described in detail and proposed to stabilise the tetramer and filament, yet none of these interactions are validated biochemical studies. Without at least key point mutations at these interfaces, the functional importance of these contacts remains assertion rather than demonstration. Are there any in vivo data to support this claim?

Author response: We thank the reviewer for raising this important point. We agree that interface-disrupting mutations would be valuable if our model depended on filament assembly as the primary mechanism of autoinhibition. However, based on our revised structural interpretation together with the new biochemical data, we now conclude that the apo ZNFX1 exists as a concentration-dependent mixture of autoinhibited monomers and tetramers/filaments, with the blocking helix—not the oligomerization interfaces—serving as the primary determinant of autoinhibition.

Accordingly, we have revised the manuscript to clarify that the ARM–ARM, 1Z–1Z, and ARM–ZnF interfaces primarily mediate tetramerization and filament assembly rather than autoinhibition itself. We also analyzed the conservation of these interfaces and found that residues mediating the ARM–ARM and 1Z–1Z interfaces are highly conserved among ZNFX1 homologues, whereas those comprising the ARM–ZnF interface show more limited conservation (revised **Supplementary Fig. 9**). Because our revised model no longer attributes autoinhibition to these oligomerization interfaces, we did not pursue additional interface-disrupting mutagenesis in the present study.

Major Point 4. While ARM–dsRNA binding has been observed in a cellular context (Wang et al. 2019, ref 12), the specific binding mode and residues proposed here (K143, K147, K180) rest entirely on an AlphaFold3 prediction. No in vitro binding data are provided in this study to validate the predicted binding interface or confirm direct, reconstituted ARM–dsRNA interaction, given the lower resolution of the map. Since this predicted interface forms the basis of the unwinding model in Figure 4F, the gap between cell-based observation and the structural claims made here should be addressed experimentally.

Author response: We thank the reviewer for raising this important point. To experimentally validate the AlphaFold3-predicted ARM-RNA interface, we expressed and purified both the isolated ARM domain and an RNA-binding mutant (ARM-3K, in which the three predicted RNA-binding residues (Lys143, Lys147, and Lys180) were replaced with glutamate residues.

We then performed AlphaScreen RNA-binding assays using both dsRNA and ssRNA substrates. The wild-type ARM domain bound both dsRNA and ssRNA, with a higher affinity for ssRNA. In contrast, the ARM-3K mutant showed no detectable binding to either substrate. These new biochemical data provide direct experimental support for the

AlphaFold3-predicted RNA-binding interface. These results have been incorporated into the revised **Fig. 5b,** and the corresponding text has been revised accordingly.

Major Point 5. The proposed unwinding mechanism (Figure 4F) lacks experimental support. It depends on the unvalidated dsRNA–ARM interaction (Major Point 4), an assumed "pulling force" generated by ssRNA translocation, and analogy to the Sen1 helicase. No unwinding assays are presented and there is no evidence that ZNFX1 can simultaneously engage ssRNA through the helicase core and dsRNA through the ARM domain. Presenting this as a concluding mechanistic figure overstates the data as it has been presented. This should either be supported with biochemistry, removed or and discussed with appropriate caveats.

Author response: We thank the reviewer for this important comment. To provide experimental support for the proposed RNA unwinding mechanism, we performed both additional structural modeling and biochemical analyses.

First, we used AlphaFold3 to predict the structure of a ZNFX1 monomer comprising the ARM domain and helicase core bound to an RNA-junction substrate containing a duplex region with a 5′ ssRNA overhang. In the resulting model, the dsRNA region engages the same ARM surface identified in the predicted ARM-dsRNA complex, whereas the 5′ ssRNA overhang extends into the RNA-binding channel formed by the helicase core and the 1C domain. This model provides a plausible structural framework in which ZNFX1 simultaneously engages dsRNA through the ARM domain and ssRNA through the helicase core.

Second, we directly examined the dsRNA unwinding activity of ZNFX1 using this RNA-junction substrate. Wild-type ZNFX1 efficiently unwound the substrate in a time- and ATP-dependent manner. The Walker A mutant (K625A) exhibited little unwinding activity, confirming that RNA unwinding depends on ATP hydrolysis. Furthermore, deletion of the ARM domain or mutation of its RNA-binding surface significantly reduced unwinding activity, demonstrating that ARM-mediated RNA binding contributes to efficient RNA unwinding. Likewise, mutations of the positively charged RNA-binding surface within the 1C domain also impaired unwinding, supporting the functional importance of ssRNA engagement by the helicase core–1C channel.

Together, these new structural predictions and biochemical data provide experimental support for the proposed unwinding model. Accordingly, we have revised the manuscript to present this mechanism as a data-supported working model rather than a purely speculative interpretation.

Major Point 6. Both datasets have preferred orientation problems. Monomeric particles in both the apo and ssRNA-bound datasets had orientation bias. For the tetramer and dimer reconstructions, the angular distribution plots (Supplementary Figure 3A) are shown but not

discussed in the main text. The authors should comment on whether orientation anisotropy affects map quality in specific regions or any of their claims.

Author response: We thank the reviewer for raising this important point. We agree that both the tetrameric ZNFX1 and the ssRNA-bound ZNFX1 dimer exhibited some degree of preferred orientation. Nevertheless, the particle orientations covered sufficiently broad angular ranges to enable reliable 3D reconstructions.

The residual orientation anisotropy, together with conformational flexibility, likely contributed to the lower local resolution observed for the ZF motifs in the tetrameric structure. In contrast, for the ssRNA-bound ZNFX1 dimer, local refinement focused on a single protomer improved the map resolution to 3.37 Å, enabling reliable atomic model building of the helicase core, RNA binding site, and dimerization interfaces.

Accordingly, we have revised the manuscript to discuss the angular distributions, their impact on local map quality, and the resulting limitations. Although preferred orientation affects the local resolution of some flexible regions, we don’t believe that it alters the principle structural conclusions of this study.

Minor Concerns

Minor Point 1. The main text reports ZNFX1 concentrations as “~20 nM” and then “0.02 to 0.2 µM” in the same passage, while Supplementary Figure 2 uses nM throughout. Units should be consistent.

Author response: We thank the reviewer for pointing out this inconsistency. We have revised the manuscript to report ZNFX1 concentration consistently in nM.

Minor Point 2. The Figure 1F caption describes an “atomic model” of a single ZNFX1 subunit, but the text states that ZnF1–6 was modelled by fitting an AlphaFold prediction into lower-resolution density. The caption should distinguish which regions are experimentally built and which are docked AlphaFold models, as the current wording is misleading for a non-specialist reader.

Author response: We thanks the reviewer for this helpful suggestion. We have revised the Figure 1F legend to clearly distinguish the regions that are built directly into the cryo-EM density from the ZnF1-6 region, which was modeled by fitting the AlphaFold prediction into lower-resolution density.

Minor Point 3. The comparison between the ZNFX1 blocking helix and the UPF1 RNA-binding site (Figures 1E–F) would be more effective as a direct structural superposition rather than two separate panels. Aligning the helicase cores would immediately show that

the blocking helix sits in the equivalent position to ssRNA in UPF1.

Author response: We thank the reviewer for this helpful suggestion. We have added a new panel (**Fig. 1g**) showing a direct structural superposition of the ZNFX1 helicase core and the ssRNA-bound UPF1 helicase core. This comparison clearly demonstrates that the blocking helix of ZNFX1 occupies the same position as the ssRNA bound to UPF1, providing a more direct visualization of the proposed autoinhibition mechanism.

Minor Point 4. For the ssRNA-bound dimer, C2 symmetry was imposed and local refinement used a soft-edged mask. It is unclear whether symmetry expansion was also applied here. Given the issues with the tetramer workflow (Major Point 1), the authors should clarify the exact processing steps for the dimer reconstruction.

Author response: We thank the reviewer for this helpful suggestion. We have applied the revised data-processing workflow to the ssRNA-bound ZNFX1 dataset using the same symmetry expansion, signal subtraction, and local refinement procedure adopted for the apo ZNFX1 tetramer/filament dataset. The revised workflow yielded a modest improvement in the final map resolution to 3.37 Å. We have updated the Methods section and the corresponding data-processing figures to accurately describe the revised reconstruction procedure.

Minor Point 5. The Methods section states a defocus range of "–1.2 to –1.0 µm" while Supplementary Table 1 reports "–1.2 to –1.8 µm." These should be reconciled.

Author response: We thank the reviewer for catching this inconsistency. We have corrected the defocus range in the Methods section to "–1.2 to –1.8 µm", consistent with Supplementary Table 1.

Minor Point 6. The filament architecture is described as "reminiscent of a double-stranded arrangement." This could be misread as referring to double-stranded nucleic acid rather than a double-helical filament. Consider rewording to "double-helical arrangement" or similar.

Author response: We thank the reviewer for this helpful suggestion. We have adopted the reviewer's recommendation and revised the wording to "double-helical arrangement" to avoid potential confusion with double-stranded nucleic acids.

Point-by-point responses to the reviewers' comments

Reviewer #1 (Remarks to the Author)

My concerns are adequately addressed. I recommend accepting this work now.

Author response: We sincerely thank the reviewer for the constructive and positive comments throughout the review process, which have helped us to improve the manuscript. We also thank their recommendation for acceptance.

Reviewer #2 (Remarks to the Author)

The authors have responded appropriately to the reviewer feedback, improving their paper by adding biochemical experiments to support their claims, focusing their conclusions on their data and increasing the detail of the methods sections. I now support publication of this manuscript in Nature Communications.

Author response: We sincerely thank the reviewer for the positive comments and support for publication of our manuscript in Nature Communications.

Reviewer #3 (Remarks to the Author)

I thank the authors for a thorough revision. The added biochemistry, comprising ATPase assays, AlphaScreen binding assays, and the first direct demonstration of ATP-dependent RNA unwinding by purified human ZNFX1, addresses my central concern, and the cryo-EM processing has been corrected as requested.

On my previous points: Major Point 1 (symmetry expansion and local refinement) is fully resolved, and the revised workflow, particle counts and map resolutions are internally consistent. Major Points 4 and 5 are substantively addressed. Major Point 3 is not tested experimentally, but the model has been restructured so that it no longer depends on those interfaces, which I accept. Major Point 6 is now discussed in the text as I asked, though a quantitative anisotropy metric would still be preferable. All six minor points are fixed. I have two remaining concerns.

Author response: We sincerely thank the reviewer for the careful evaluation of our revised manuscript and for the positive comments on the additional biochemical experiments and cryo-EM analyses.

1. Modelled contacts are presented as observed contacts. The Methods state that the 1Z-1Z interface was built from an AlphaFold prediction because the density was insufficient, and the Results that the zinc-finger region was fitted into lower-resolution density. That disclosure is not carried through to the claims: both interfaces are then described in the same register as the ARM-ARM interface, with individual contacts named as though

observed, marked as interface residues in the alignment, and used for a conservation contrast in the Discussion. Of the three, only the ARM-ARM interface is well resolved and independently corroborated by the published ARM dimer crystal structure. Please identify the predicted contacts as such wherever they are asserted and temper the conservation argument accordingly.

Author response: We thank the reviewer for this helpful comment. Following the reviewer's suggestion, we have revised both the results and discussion sections to clearly distinguish the levels of structural support for the three different interfaces. The ARM–ARM interface is well resolved and independently supported by the reported ARM dimer crystal structure. Although the overall 1Z density was insufficient for unambiguous de novo modeling, density was present at the 1Z–1Z interface and closely matched the AlphaFold3-predicted model. The less well-resolved ARM–ZnF interface and its proposed contacts are now explicitly identified as putative and model-based, indicating that the proposed charge–charge interactions should be interpreted with caution.

2. The functional dissection needs more cautious framing. Deleting the ARM domain and mutating its RNA-binding surface each reduce unwinding only modestly, and indistinguishably from one another and from the less extensively substituted 1C variant; only the Walker A and most extensively substituted 1C mutants impair it severely. This indicates a contributory rather than obligatory role for the ARM with this short junction substrate, so the stated requirement for engagement by both the ARM and the helicase core overstates the data; "progressively" is likewise unsupported for the 1C series. The two ARM constructs also appear to be assigned their values in opposite order in the text and the figure key.

Author response: We thank the reviewer for this careful assessment. We agree that our original interpretation overstated the requirement for the ARM domain. We have revised the text to indicate that the ARM domain contributes to, but is not essential for, RNA unwinding with the short junction substrate used here. Accordingly, we now state that coordinated ATP hydrolysis and RNA engagement involving the ARM domain and helicase core contribute to efficient unwinding, rather than being strictly required. We have also removed "progressively" from the description of the 1C mutants, reported their effects individually. In addition, we have tempered the final mechanistic model by proposing that the ARM domain may engage and stabilize structured RNA. Finally, we thank the reviewer for identifying this discrepancy and have corrected the values for the two ARM constructs in the text and figure key.
